# Development and validation of allele-specific SNP/indel markers for eight yield-enhancing genes using whole-genome sequencing strategy to increase yield potential of rice, *Oryza sativa* L.

**DOI:** 10.1186/s12284-016-0084-7

**Published:** 2016-03-18

**Authors:** Sung-Ryul Kim, Joie Ramos, Motoyuki Ashikari, Parminder S. Virk, Edgar A. Torres, Eero Nissila, Sherry Lou Hechanova, Ramil Mauleon, Kshirod K. Jena

**Affiliations:** Plant Breeding, Genetics, and Biotechnology Division, International Rice Research Institute, Metro Manila, Philippines; Bioscience and Biotechnology Center, Nagoya University, Nagoya, Japan; CIAT-HarvestPlus, 303 ICRISAT Campus, Patancheru, Telungana India; International Center for Tropical Agriculture, A.A. 6713 Cali, Colombia

**Keywords:** Rice, Yield potential, Yield-enhancing genes, Allele-specific marker, SNP genotyping

## Abstract

**Background:**

Rice is one of the major staple foods in the world, especially in the developing countries of Asia. Its consumption as a dietary source is also increasing in Africa. To meet the demand for rice to feed the increasing human population, increasing rice yield is essential. Improving the genetic yield potential of rice is one ideal solution. It is imperative to introduce the identified yield-enhancing gene(s) into modern rice cultivars for the rapid improvement of yield potential through marker-assisted breeding.

**Results:**

We report the development of PCR-gel-based markers for eight yield-related functional genes (*Gn1a*, *OsSPL14*, *SCM2*, *Ghd7*, *DEP1*, *SPIKE*, *GS5*, and *TGW6*) to introduce yield-positive alleles from the donor lines. Six rice cultivars, including three each of donor and recipient lines, respectively, were sequenced by next-generation whole-genome sequencing to detect DNA polymorphisms between the genotypes. Additionally, PCR products containing functional nucleotide polymorphism (FNP) or putative FNPs for yield-related genes were sequenced. DNA polymorphisms discriminating yield-positive alleles and non-target alleles for each gene were selected through sequence analysis and the allele-specific PCR-gel-based markers were developed. The markers were validated with our intermediate breeding lines produced from crosses between the donors and 12 elite *indica* rice cultivars as recipients. Automated capillary electrophoresis was tested and fluorescence-labeled SNP genotyping markers (Fluidigm SNP genotyping platform) for *Gn1a*, *OsSPL14*, *Ghd7*, *GS5*, and *GS3* genes were developed for high-throughput genotyping.

**Conclusions:**

The SNP/indel markers linked to yield related genes functioned properly in our marker-assisted breeding program with identified high yield potential lines. These markers can be utilized in local favorite rice cultivars for yield enhancement. The marker designing strategy using both next generation sequencing and Sanger sequencing methods can be used for suitable marker development of other genes associated with useful agronomic traits.

**Electronic supplementary material:**

The online version of this article (doi:10.1186/s12284-016-0084-7) contains supplementary material, which is available to authorized users.

## Background

Food security is threatened by the growing human population and decreasing agricultural resources, including crop land and labor, through urbanization and industrialization. Current global yield increase rates (0.9-1.3% per year) of the four major crops (rice, maize, wheat, and soybean) are insufficient to meet food demand for the estimated nine billion people in 2050 (Khush 2005; Ray et al. 2013). Rice is a major staple food in the world, especially in the developing countries of Asia. In Africa, it is the most rapidly growing food source and, according to a conservative estimate, about 30 million tons more rice will be needed by 2035 (Seck et al. 2012). Within existing agricultural lands, the genetic improvement of yield potential in rice could be the ideal way to increase yield.

Three major traits (grain size, grain number per panicle, and panicle number per plant) are directly associated with rice grain productivity, and these traits strongly depend on the genetic potential of rice. However, these traits are complex and quantitative in nature. Through quantitative trait loci (QTL) analysis with fine mapping or positional cloning using rice mutants, about 20 genes that are involved in yield-related traits have been isolated in rice (Wang and Li 2005; Xing and Zhang 2010; Miura et al. 2011; Huang et al. 2013; Liu et al. 2015). In regards to the genes controlling grain size, six genes can be considered in rice breeding programs. The *GW2* encoding RING-type E3 ubiquitin ligase regulates grain width. The loss of GW2 function by a premature stop codon caused by a 1-bp deletion on the fourth exon increased grain width, resulting in increased yield (Song et al. 2007). The *GS3* gene encoding a putative transmembrane protein functions as a negative regulator for grain size. The nucleotide substitution from **C** to **A** on the second exon resulted in the stop codon (TG**A**) and eventually caused C-terminal truncated GS3 protein. This mutated allele increased grain size (Fan et al. 2006). The *qSW5*/*GW5* gene encoding a nuclear-localized unknown protein is involved in the regulation of grain width. The loss-of-function allele by a 1.2-kb deletion including coding DNA sequence (CDS) of the gene increases grain width, and this allele is common in *japonica* varieties (Shomura et al. 2008; Weng et al. 2008). The *GS5* gene encoding a putative serine carboxypeptidase regulates grain size. Different expression level of *GS5* based on promoter sequences is associated with grain width (Li et al. 2011). *OsSPL16*/*qGW8*, encoding SQUAMOSA promoter binding protein-like (SPL) 16, controls grain width, and higher expression in young panicle promotes grain width (Wang et al. 2012). The *TGW6* gene encoding a novel protein having indole-3-acetic acid (IAA)-glucose hydrolase activity regulates grain weight. The mutant allele (1-bp deletion in CDS) showed increased thousand-grain weight (Ishimaru et al. 2013). Among the genes regulating grain number per panicle, several seem useful in a rice breeding program. The *Gn1a* gene encoding cytokinin oxidase/dehydrogenase2 (OsCKX2) regulates grain number per panicle. The non-functional allele of *Gn1a* increased grain number per panicle, resulting in increased yield (Ashikari et al. 2005). The *Ghd7* encoding a CCT domain protein is involved in the regulation of heading date, plant height, and grain number per panicle. Fully functional alleles *Ghd7-1* and *Ghd7-3* showed delayed heading date and increased plant height and yield (Xue et al. 2008). The *DEP1* gene encoding a phosphatidylethanolamine-binding protein-like domain protein regulates panicle architecture. The mutant allele produced short panicle with high grain number per panicle, resulting in increased yield (Huang et al. 2009). The *SPIKE/NAL1* gene encoding an unknown function protein regulates grain number per panicle. Either transcription level or three amino acids changes on NAL1 protein are associated with phenotype (Fujita et al. 2013). The *SCM2*/*APO1* encoding F-box-containing protein controls grain number per panicle and culm diameter. Abundant transcripts of *SCM2* in developing panicles increased grain number (Ookawa et al. 2010). Higher expression of *OsSPL14*/*WFP*/*IPA1* gene encoding OsSPL14, in young panicle is associated with higher grain number per panicle (Jiao et al. 2010; Miura et al. 2010). Regarding the genes regulating panicle number per plant, several have been identified through positional cloning from the rice monoculm mutant *moc1* and rice dwarf mutants with higher tiller number (Jeon et al. 2011). However, the reported alleles of these genes may not be desirable in actual breeding programs because of too much reduction in tiller number of *moc1* mutant or dwarfism in the high tiller number mutants.

Some PCR markers for the yield-related genes were developed and used for marker-assisted breeding and gene function study. In the *GS3* gene, the C/A single nucleotide polymorphism (SNP) on the second exon regarded as a functional nucleotide polymorphism (FNP) is directly associated with the phenotype, and it can be discriminated by digestion of PCR product with *Pst*I restriction enzyme. Cleaved amplified polymorphic sequence (CAPS) markers for C/A SNP were reported (Fan et al. 2009; Yan et al. 2009; Wang et al. 2011). Additionally, Wang et al (2011) developed gene-tagged markers using the difference in repeat number of di- or tri-nucleotides within *GS3* for marker-assisted selection (MAS). In the *GW2* gene, the 1-bp deletion on the fourth exon is the FNP. This mutation was detected by the CAPS marker using *Hpa*I restriction enzyme (Yan et al. 2009; Yan et al. 2011). For *qSW5*/*GW5* gene, three alleles (Nipponbare type, Kasalath type, and *indica* II type) were discriminated by the N1212 marker, which used the difference in PCR product sizes among the alleles (Shomura et al. 2008; Yan et al. 2011). For the genes regulating grain number per panicle, the markers of *Gn1a*, *OsSPL14*, and *DEP1* genes were reported. Yan et al (2009) developed the Gn1a-M1 marker for the detection of *Gn1a*-Habataki allele, which has a 16-bp deletion in the 5’-untranslated region (5’UTR), and the Gn1a-M2 marker for the detection of *Gn1a*-5150 allele, which has an 11-bp deletion on the third exon. Xu et al (2014) developed the CAPS marker using *Sdu*I restriction enzyme to select *OsSPL14*-Ri22 allele, which transcribes OsmiR156-resistant *OsSPL14* transcripts caused by the nucleotide substitution at the OsmiR156 target site. They also developed the gene-tagged PCR marker of *DEP1* gene using the 625-bp gap on the fifth exon between the yield-positive allele and non-target allele.

The allele-specific gene-tagged markers for the target genes are more effective than the genomic random markers surrounding the target gene (from several kb to a few Mb distance) because some markers will not show polymorphism in some recipient backgrounds and sometimes a false-positive allele can be selected by recombination between the target gene and the genomic random marker. However, allele-specific markers for yield-enhancing genes have not been developed yet for some major yield-enhancing genes or the existing markers need to be improved for an effective marker-assisted breeding program. The allele-specific gene-tagged markers were well developed for *GS3*, *GW2*, and *qSW5*/*GW5* genes as mentioned above. But, for *GS5*, *TGW6*, *SCM2*, *Ghd7*, and *SPIKE* genes, PCR markers for selecting the yield-positive alleles have not been developed yet. Additionally, the marker that can select the yield-positive *OsSPL14*-ST12 allele, which is different from the *OsSPL14*-Ri22 allele, has not been reported. For the *Gn1a* gene, a 16-bp deletion in 5’UTR is common in *indica* rice varieties (54.3%) (Yan et al. 2009), suggesting that the *Gn1a*-M1 marker may not function in many *indica* varieties. For the *DEP1* marker (Xu et al. 2014), the intensity of the non-target allele band was very weak compared with that of the target allele in the heterozygous plants. To obtain clear genotype data, the previous *DEP1* marker needs to be improved.

Although around 20 yield-related genes have been identified in rice, their evaluation and use are still limited for rice breeding programs. These studies are very important for improving the genetic yield potential of rice effectively. To evaluate and use the yield enhancing genes, in this study we report allele-specific gene-tagged markers for eight yield-related genes. For effective screening of DNA polymorphisms of yield-enhancing genes, we employed both next-generation sequencing as well as Sanger sequencing methods. For easy access and user-friendly markers, we developed PCR-gel-based markers without restriction enzyme digestion. In addition, for high-throughput genotyping using a fluorescence detection system, we also developed Fluidigm SNP genotyping markers. Moreover, the allele-specific PCR-gel based markers were validated through its application in our marker-assisted selection (MAS) breeding program.

## Results and Discussion

Development of allele-specific markers for eight yield-enhancing genes was conducted as a part of our breeding program for increasing genetic yield potential in 12 elite *indica* varietal backgrounds (Table 1). For determination of alleles of yield-enhancing genes in 12 recipient cultivars and effective screening of polymorphisms between the donor and the recipient, we sequenced the whole genomes of three donors (Habataki, ST6, and ST12) and three recipients (NSIC Rc158, NSIC Rc222, and NSIC Rc238) using next-generation sequencing technique. In addition, the target yield-enhancing genes were also sequenced from the 12 recipients and the donors using Sanger sequencing method.

**Table 1:** The donor cultivars of yield-enhancing genes and 12 recipients

Variety	Class	Origin	Donor/recipient
Habataki^a^	*indica*	Japan	*Gn1a*, *SCM2*, *Ghd7*
ST12^a^	*indica*	Japan	*Gn1a*, *OsSPL14*
ST6^a^	*japonica*	Japan	*Gn1a*, *GS5*
Aikawa1	*japonica*	Japan	*OsSPL14*
YP9^b^	*indica*	Philippines	*SPIKE*
Osmancik-97	*japonica*	Turkey	*DEP1*
Kasalath	*indica*	India	*TGW6*
PSB Rc82	*indica*	Philippines	recipient
NSIC Rc158^a^	*indica*	Philippines	recipient
NSIC Rc222^a^	*indica*	Philippines	recipient
NSIC Rc238^a^	*indica*	Philippines	recipient
IR04A115	*indica*	Philippines	recipient
IR05N412	*indica*	Philippines	recipient
PR37951-3B-37-1-2	*indica*	Philippines	recipient
PR38012-3B-3-1	*indica*	Philippines	recipient
CT5803	*indica*	Colombia	recipient
CT5805	*indica*	Colombia	recipient
Irga427	*indica*	Colombia	recipient
Parao	*indica*	Colombia	recipient

^a^Entry of whole-genome sequencing

^b^Near-isogenic line (*SPIKE*-Daringan allele in IR64 genetic background)

Generally, several DNA polymorphisms such as indel and/or SNP were found between the two parental alleles as a result of fine mapping of major QTLs. Among polymorphisms, the FNP was identified in some yield-enhancing genes such as *GS3*, *GW2*, *DEP1*, and *TGW6* genes but not in some other genes. For marker designing, firstly we targeted FNP or putative FNPs of yield-related genes. Secondly, we selected the donor-specific polymorphisms within the target gene to select the donor allele easily. Actually, when breeders have the original donor source which was used for QTL/fine-mapping analysis, any DNA polymorphisms within or near the target gene between the donor and the recipient can be used to design a marker for MAS (Usually indel polymorphisms are preferred to design markers because of convenience to marker designing and clear genotype results by the markers). The selected polymorphisms for each yield-related gene and its corresponding markers were described below one by one. Finally, the markers we developed were validated through its application in our MAS breeding lines.

### *Gn1a* Markers

**Fig. 1:**
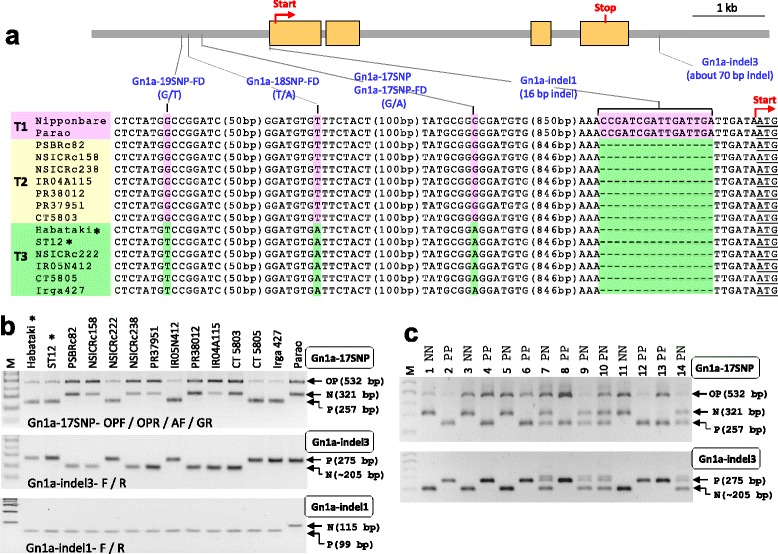
*Gn1a* markers. **a** Identification of nucleotide polymorphisms in *Gn1a* gene for marker development. *Gn1a* gene consists of four exons (*orange boxes*) in which translation initiation codon (*Start*) and stop codon (*Stop*) are depicted. Each nucleotide variation with corresponding markers (PCR-gel-based markers and Fluidigm genotyping platform (-FD) markers) is mapped on the gene structure. The sequence alignment showed nucleotide polymorphisms in the promoter and 5’UTR regions of *Gn1a* among 15 varieties, including the reference genome (Nipponbare), two *Gn1a* donors, and 12 recipients. DNA polymorphisms were highlighted with pink and green color. The number of unrepresented nucleotides (bp) in a sequence was shown in parentheses. Based on the context of the promoter sequences, three *Gn1a* alleles (Types 1-3: T1-T3) were found. Variety name with asterisk (*) was used as the donor line of target allele. **b** Agarose gel images analyzed by three *Gn1a* markers (Gn1a-17SNP, Gn1a-indel3, and Gn1a-indel1 markers) from two donor lines and 12 recipients. Predicted PCR product sizes for yield-positive allele (P), non-target allele (N), and common band (OP) were shown at the right side of the gel image. Primer combination for each marker was shown on the gel images and its sequences were listed in Table 2. M, DNA size marker. **c** Application of *Gn1a* markers in the intermediate breeding line. Fourteen BC_1_F_3_ plants derived from PR37951 x Habataki cross were genotyped with Gn1a-17SNP and Gn1a-indel3 marker, respectively. Genotyping result was scored for each plant as PP (homozygous for positive allele), NN (homozygous for non-target allele), and PN (heterozygote)

Yield-positive *Gn1a* allele was originally isolated from the high-yielding rice variety Habataki through QTL analysis with positional cloning (Ashikari et al. 2005) and *Gn1a*-Habataki allele was also found in cultivar ST12 (Miura et al. 2010). These two materials were used as a *Gn1a* donor in our marker-assisted breeding program. We screened DNA variations in the *Gn1a* gene, including in the 5-kb upstream region from the start codon and 2-kb downstream region from the stop codon, using IGV software (Robinson et al. 2011) with WGS data. Within this range, the sequences were the same among Habataki, ST6, ST12, and NSIC Rc222, indicating that these four accessions have the yield-positive *Gn1a* allele. Three SNPs in the promoter region were found based on WGS analysis (Additional file 1: Figure S1A) and the sequences of three SNPs were confirmed by Sanger sequencing of PCR products from *Gn1a* donors and 12 recipients (Fig. 1a). Based on the sequence analysis, all genotypes were divided into three groups (Types 1-3; Fig. 1a). NSIC Rc222, IR05N412, CT5805, and Irga427 among the 12 recipients have *Gn1a*-Habataki allele (Type 3). First, we developed the Gn1a-indel1 marker to detect a 16-bp indel in 5’UTR as identified by Ashikari et al (2005). Except for Parao, all genotypes showed the same band size as Habataki (Fig. 1b bottom), indicating that this marker is available only for *Gn1a* MAS in a Parao background. Next, we developed the Gn1a-17 SNP marker using the tetra-primer PCR method (Additional file 2: Figure S2A) to discriminate G/A SNP in the promoter region (Fig. 1a). The marker discriminated G/A SNP clearly in the parents (Fig. 1b top) as well as in intermediate breeding lines that contained heterozygous plants (Fig. 1c). This marker is available to introduce *Gn1a*-Habataki allele (Type 3) into both *Gn1a*-Type 1 and *Gn1a*-Type 2 backgrounds. Additionally, we developed an indel-type marker, the Gn1a-indel3 marker, based on WGS data analysis, which showed approximately 70-bp indel polymorphism near 3’UTR (Additional file 1: Figure S1B). This marker was tested in the parental varieties, and the result was consistent with the Gn1a-17 SNP marker analysis, except for Parao (Fig. 1b middle), indicating that the marker is available to introduce *Gn1a*-Habataki allele into *Gn1a*-Type 2 backgrounds but not Parao background. The Gn1a-indel3 marker was also applied to intermediate breeding lines and the genotyping results were consistent with those of the Gn1a-17 SNP marker (Fig. 1c). Based on the *Gn1a* sequence context of the background varieties, a suitable marker among the Gn1a-indel1, Gn1a-indel3, and Gn1a-17 SNP markers can be used to select the yield-positive *Gn1a* allele.

Reduced expression of *Gn1a* in inflorescence meristem of Habataki elevates cytokinin content, resulting in high grain number per panicle. Therefore, we tried to find polymorphisms in the *Gn1a* promoter region, which is the regulatory region for gene transcription. Through WGS and PCR product sequencing of the *Gn1a* gene, we found three SNPs in the promoter region. It was presumed that any of the three SNPs were associated with *Gn1a* expression level. Based on the combination of the three SNPs and 16-bp indel in the *Gn1a* promoter region, we assigned rice accessions into three *Gn1a* alleles (Types 1-3) (Fig. 1a). Earlier the effect of the *Gn1a*-Habataki allele was evaluated in only *japonica* backgrounds (Type 1) (Ashikari et al. 2005; Ohsumi et al. 2011). When 35 *indica* varieties were analyzed by the *Gn1a*-M1 marker that discriminates the 16-bp indel in the 5’UTR, 19 accessions (54.3%) showed a Habataki band pattern (Yan et al. 2009), suggesting that these lines are Type 2 or Type 3 alleles. In our analysis, 11 out of 12 recipients showed 16-bp deletion in the 5’UTR like Habataki. This suggested that many *indica* varieties belong to Type 2 or Type 3 alleles, unlike *japonica* varieties. However, the effect of the *Gn1a*-Habataki allele (Type 3) needs to be tested in Type 2 backgrounds through the development of near-isogenic lines (NILs). If the Type 3 allele is functionally the same as the Type 2 allele (i.e., no grain number difference between the recipient variety and NIL-Type 3), rice breeders do not need to introduce the *Gn1a*-Habataki allele into Type 2 cultivars. The previous Gn1a-M1 marker is not available to introduce the Habataki allele in Type 2 backgrounds because it cannot discriminate the Type 2 allele and Type 3 allele. The Gn1a-17SNP marker and three Fluidigm SNP markers (Gn1a-17SNP-FD, Gn1a-18SNP-FD, and Gn1a-19SNP-FD) are effective for introducing the *Gn1a*-Habataki allele into *Gn1a*-Type 1 background as well as Type 2 background. Regarding detection of the 16-bp indel, we newly designed the Gn1a-indel1 marker, which enhanced the band separation in agarose gel by reducing PCR product sizes (99 bp and 115 bp) compared with those of the Gn1a-M1 marker (113 bp and 129 bp). The Gn1a-indel3 marker located near the 3’UTR was developed for obtaining clear genotype data. The marker showed clear band separation in agarose gel and similar PCR efficiency between the two alleles in heterozygotes (Fig. 1c). Although the Gn1a-indel3 marker was not associated with the phenotype, it will be helpful for MAS of the *Gn1a* gene in many *indica* cultivar backgrounds.

### *OsSPL14*/*WFP*/*IPA1* Markers

**Fig. 2:**
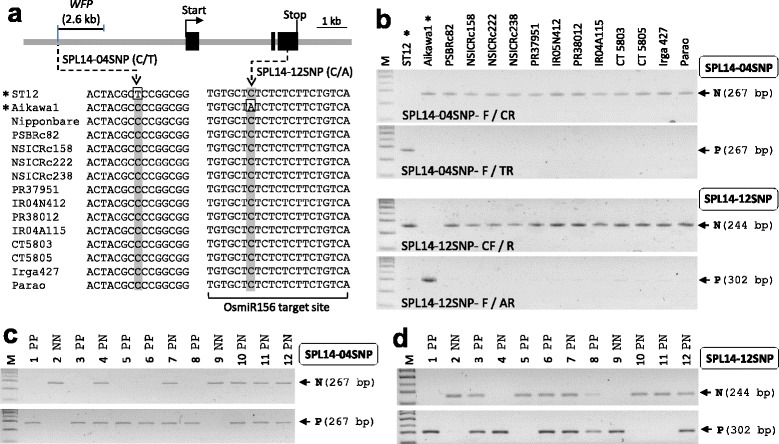
*OsSPL14* markers. **a** Identification of donor-specific DNA polymorphisms in *OsSPL14* gene. The donor-specific SNPs at the promoter and the third exon were represented through DNA sequence alignment among 15 varieties, including Nipponbare, two different *OsSPL14* donors (ST12 and Aikawa1), and 12 recipients. The location of 2.6-kb candidate region of *WFP* (Miura et al. 2010) was depicted. **b** Agarose gel images analyzed by the SPL14-04SNP marker and the SPL14-12SNP marker, respectively, from the parental lines. Primer combination for each PCR was shown on the gel images. **c** Application of SPL14-04SNP marker in the intermediate breeding line, with 12 BC_2_F_2_ plants derived from PR38012 x ST12 cross. **d** Application of SPL14-12SNP marker in the intermediate breeding line. Twelve BC_2_F_2_ plants derived from IR04A115 x Aikawa1 cross were genotyped

The QTL *WFP* (*WEALTHY FARMER’S PANICLE*) encoding OsSPL14 negatively regulates the number of tillers in the vegetative stage and positively controls the number of rachis in the reproductive stage. Higher expression of *OsSPL14* in young panicles increased panicle branching and grain number per panicle, resulting in increased yield. Two different yield-positive alleles (*OsSPL14*-ST12 and *OsSPL14*-Aikawa1) showed higher expression in inflorescence with different mechanisms. In the line ST12, *OsSPL14* transcripts were abundant by less DNA methylation in the *OsSPL14* promoter region compared with that of Nipponbare rather than by DNA sequence differences (Miura et al. 2010). The *OsSPL14*-ST12 allele belongs to epigenetic alleles that show heritable gene expression difference which is caused not by DNA sequence variations but by DNA methylation or chromatin status (Kakutani 2002). In contrast, the *OsSPL14*-Aikawa1 allele expressed OsmiR156-resistant *OsSPL14* transcripts because of the nucleotide substitution (C to A) at the OsmiR156 target site located on the third exon of *OsSPL14*, resulting in higher expression in panicles (Miura et al. 2010). The same alleles with Aikawa1 were isolated through fine mapping of QTL *IPA1* (*Ideal Plant Architecture 1*) from the *japonica* lines Shaoniejing and Ri22 (Jiao et al. 2010). Through WGS data analysis, we found a putative ST12-specific SNP (C/T) in the *OsSPL14* promoter region (Additional file 3: Figure S3). Finally, this was confirmed by direct sequencing of PCR product from line ST12 and 12 recipients (Fig. 2a). The SNP was selected for developing the SPL14-04SNP marker which consists of two PCRs: the C-allele primer set (SPL14-04SNP-F/CR) and the T-allele primer set (SPL14-04SNP-F/TR). The SPL14-04SNP marker showed allele-specific PCR amplification in parental lines (Fig. 2b) and in intermediate breeding lines harboring heterozygous plants (Fig. 2c). The Aikawa1-specific SNP located at OsmiR156 target site was confirmed by Sanger sequencing from Aikawa1 and 12 recipients (Fig. 2a). The SNP was used for developing the SPL14-12 SNP marker to select *OsSPL14*-Aikawa1 allele. The non-Aiakawa1 allele and Aikawa1 allele were amplified with SPL14-12SNP-CF/R primer set and SPL14-12SNP-F/AR primer set, respectively. The marker discriminated the alleles clearly from the parents (Fig. 2b) and intermediate breeding lines (Fig. 2d).

The *OsSPL14*-ST12 allele is an epigenetic allele. Less DNA methylation in *OsSPL14* promoter in ST12 increased its transcription, resulting in high grain number per panicle. However, we can introduce the *OsSPL14*-ST12 allele using DNA polymorphism within or near the *OsSPL14* gene between ST12 and the recipient instead of checking the status of DNA methylation. Among the six varieties analyzed by WGS, Habataki had many unique DNA polymorphisms (18 SNPs and one 2-bp deletion) in the *OsSPL14* gene located on chromosome 8 (data not shown). But, the new *OsSPL14* allele, Habataki allele, may not be a new yield-positive allele because the QTL controlling grain number per panicle was not detected on chromosome 8 (Ashikari et al. 2005). However, we found an ST12-specific C/T SNP in the promoter region, which was about 4.2 kb distance from the start codon. This nucleotide was identical to the border nucleotide of the 2.6-kb *WFP* candidate region (Miura et al. 2010), indicating that the SNP can be an FNP of the *OsSPL14*-ST12 allele. In plants, DNA methylation occurs through the addition of a methyl group to the cytosine bases of DNA in the context of CG, CHG, and CHH (H = A, C, or T) (He et al. 2011). The nucleotide substitution from **C** to **T** resulted in **C**CC (other varieties) and **T**CC (ST12) sequence context, respectively (Fig. 2a). If this **C** base is a target site of methylation, the **T** base of ST12 can avoid methylation, indicating higher *OsSPL14* expression. Miura et al (2010) tested DNA methylation levels in the 2.6-kb *WFP* candidate region using bisulfite sequencing and the result showed some differences in the region between ST12 and Nipponbare, which is about 1070-bp distance from the C/T SNP locus. Further study is needed to show that this SNP has some function.

The marker for the *OsSPL14*-ST12 allele has not been reported. We developed the SPL14-04SNP marker using the ST12-specific SNP to select the *OsSPL14*-ST12 allele. This marker will be available for most *indica* and *japonica* background varieties because the SNP is quite unique in ST12. To select *OsSPL14*-Aikawa1/Shaoniejing/Ri22 allele, Xu et al. (2014) developed the CAPS marker using *Sdu*I restriction enzyme. Here, we introduced the allele-specific PCR-gel-based marker, the SPL14-12SNP marker. This marker will be effective for MAS to most of rice varieties because the *OsSPL14*-Aikawa1 allele is rare.

### *SCM2*/*APO1* Marker

**Fig. 3:**
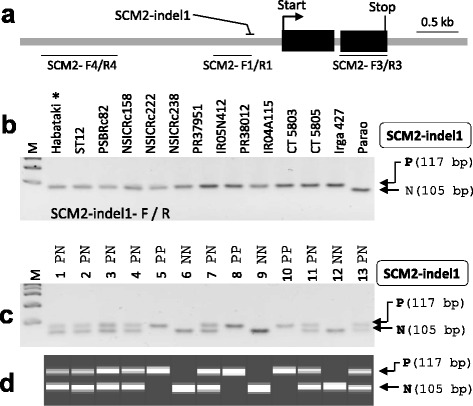
*SCM2* marker. **a** Gene structure of *APO1*/*SCM2*. Three regions sequenced by the Sanger method were represented with primer pairs. **b** Agarose gel image analyzed by SCM2-indel1 marker from the donor line (Habataki), ST12, and 12 recipients. To separate two bands (117 bp and 105 bp) produced by the SCM2-indel1 marker, 4% agarose gel was used. **c-d** Application of SCM2-indel1 marker in the intermediate breeding line. Thirteen BC_3_F_2_ plants derived from Parao x Habataki cross were genotyped by the SCM2-indel1 marker and its PCR products were electrophoresed in 4 % agarose gel (**c**) and in Fragment Analyzer (**d**)

*ABERRANT PANICLE ORGANIZATION1* (*APO1*) encodes an F-box-containing protein and is involved in controlling rachis branching in panicle, tiller outgrowth, and culm diameter. The *apo1* mutant formed smaller inflorescences with reduced numbers of branches and spikelets (Ikeda et al. 2007). In contrast, the *APO1*-overexpressing mutant, *Undulate rachi1* (*Ur1*) and *APO1*-overexpressing transgenic plants showed dramatic increment of grain number per panicle but tiller number per plant reduced remarkably (Murai and Iizawa 1994; Ikeda-Kawakatsu et al. 2009). *STRONG CULM2 (SCM2)* is a mild allele of *APO1* found in variety Habataki (2-3 times higher expression of *APO1* in inflorescence than that of Koshihikari) and the allele increased culm diameter and grain number per panicle without a reduction in tiller number (Ookawa et al. 2010; Terao et al. 2010). This *SCM2-*Habataki allele is regarded as a useful allele of *APO1* gene for increasing yield and lodging resistance in a breeding program. Through WGS data analysis, we tried to find Habataki-specific DNA variations in the *SCM2* gene (-5 kb from start codon and +2 kb from stop codon) among the six cultivars. However, no sequence difference was found among the five genotypes (Habataki, ST12, NSIC Rc158, NSIC Rc222, and NSIC Rc238). To determine the *APO1* allele from 12 recipients, we sequenced two promoter regions and the third exon (Fig. 3a), which showed some DNA variations between genotypes of mapping bi-parents (Habataki and Koshihikari) (Ookawa et al. 2010). Except for Parao, 11 recipients had the same sequence as Habataki. However, the 12-bp indel in the promoter region (Fig. 3a) was selected for marker development to introduce the *SCM2*-Habataki allele into Parao. We developed the SCM2-indel1 marker and tested it in the parental lines. As expected, only Parao showed a different band size (Fig. 3b). The marker was also applied in intermediate breeding lines that were BC_3_F_2_ plants derived from a Parao x Habataki cross and it distinguished the genotypes clearly (Fig. 3c). For automated high-throughput electrophoresis, the same PCR product was analyzed using Fragment Analyzer (Fig. 3d).

The WGS and PCR product sequencing showed that the *SCM2*-Habataki allele was detected in most of our donors and recipients. These results indicate that the high-yielding and lodging-resistant *SCM2*-Habataki allele is widely dispersed in *indica* varieties. In contrast, *temperate japonica* varieties Nipponbare, Koshihikari, Sasanishiki, and Aikawa1 had the non-yield-positive *SCM2* allele, suggesting that the *SCM2*-Habataki allele will be effective in many *temperate japonica* varieties. The *SCM2*-indel1 marker may be helpful for MAS in *japonica* backgrounds. The *SCM2* sequence of ST6 was almost the same as that of Nipponbare but two 2-bp deletions in the promoter region (3.4-kb and 5.8-kb regions from the start codon) were found in only the line ST6 among the six lines in the WGS analysis carried out. The new allele, *SCM2*-ST6, needs to be tested if the ST6 allele is superior to the Habataki allele through checking *SCM2* expression level in stem, young panicle, and phenotypes in NIL-ST6 because the grain number per panicle and culm diameter of ST6 were higher than those of Habataki as observed at IRRI during the 2013-2015 experiments.

### *Ghd7* Marker

**Fig. 4:**
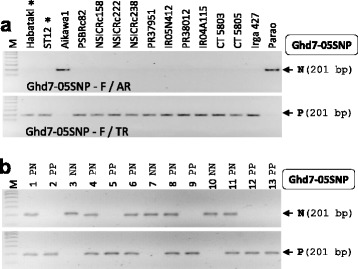
*Ghd7* marker. **a** Agarose gel image analyzed by Ghd7-05SNP marker from the parental lines. Non-target allele and yield-positive allele (*Ghd7-1*) were amplified by Ghd7-05SNP-F/AR primer pairs and Ghd7-05SNP- F/TR primer pairs, respectively. Habataki and ST12 were used as the donors of *Ghd7-1* allele and Aikawa1 (*japonica-*type variety) was included as a control of *Ghd7-2* allele. **b** Application of Ghd7-05SNP marker in the intermediate breeding line. Thirteen BC_2_F_2_ plants crossed between Parao and Habataki were genotyped by the marker

*Grain number, plant height, and heading date7* (*Ghd7*) encoding a CCT domain protein is involved in multiple yield-related traits, including heading date, plant height, and grain number per panicle (Xue et al. 2008). Nine *Ghd7* alleles (*Ghd7-0 ~ 7* and *Ghd7-0a*) were identified based on the predicted protein sequences from about 120 genotypes (Xue et al. 2008; Lu et al. 2012). To know the allele type of *Ghd7* from our breeding materials, we analyzed the genomic sequence (protein coding regions and an intron) of *Ghd7* using WGS data of six genotypes. ST6 variety had *Ghd7-6* allele and the others had exactly the same DNA sequences as variety Minghui63, which had the fully functional yield-positive *Ghd7-1* allele. Additionally, we sequenced PCR products containing *Ghd7* coding sequences from Habataki, ST12, ST6, and 12 recipients, resulting in that all lines (except for ST6 and Parao) had the *Ghd7-1* allele. These results indicated that 11 out of 12 recipients already have the yield-positive *Ghd7-1* allele. However, we selected the A/T SNP (521st nucleotide from ATG in CDS) and developed the Ghd7-05SNP marker detecting the SNP to introduce the *Ghd7-1* allele into Parao which had the *Ghd7-2* allele (a functional but weaker allele). Genotyping results from parental lines through this marker were consistent with DNA sequencing data, indicating that the marker discriminated A (non-target allele) and T (*Ghd7-1* allele) clearly (Fig. 4a). To replace the *Ghd7-2* allele with the *Ghd7-1* allele in the Parao background, this marker was used in intermediate breeding lines derived from a Parao x Habataki cross (Fig. 4b).

Based on our sequencing data analyses of *Ghd7*, the *Ghd7-1* allele is common in *indica* rice varieties. This result was consistent with the previous reports (Xue et al. 2008; Lu et al. 2012) that *Ghd7-1* and *Ghd7-2* alleles were popular in *indica* and *japonica* varieties, respectively. So, the fully functional *Ghd7-1* allele may be effective for increasing yield potential in many *japonica* varieties which are growing in long-day condition. The Ghd7-05SNP marker will be helpful to introduce *Ghd7-1* allele into *japonica* backgrounds.

### *DEP1* Marker

**Fig. 5:**
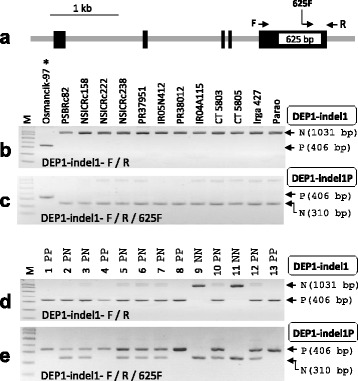
*DEP1* marker. **a** Gene structure of *DEP1*. FNP (625-bp indel) was shown with the unfilled box on the fifth exon. The location and direction of marker primers (DEP1-indel1-F/R/625F) are mapped on the gene structure. **b**-**c** Agarose gel images of PCR with DEP1-indel1 marker (**b**) and DEP1-indel1P marker (**c**) from the parents. **d**-**e** Application of DEP1-indel1 marker (**d**) and DEP1-indel1P marker (**e**) in the intermediate breeding line. Thirteen BC_2_F_2_ plants derived from NSIC Rc158 x Osmancik-97 cross were genotyped

*DENSE AND ERECT PANICLE1 (DEP1)* encodes a phosphatidylethanolamine-binding protein-like domain protein and regulates panicle architecture. The natural *DEP1* mutant allele (625-bp deletion on the fifth exon) encoding C-terminal truncated DEP1 protein increased grain number per panicle, resulting in increased yield, although panicle length decreased. This allele might be derived from Italian landrace Balilla and was distributed to many high-yielding Chinese *japonica* varieties (Huang et al. 2009). In order to detect FNP, the forward primer and the reverse primer of the DEP1-indel1 marker were located upstream and downstream of the 625-bp deletion on the fifth exon, respectively (Fig. 5a). As a result, the difference in PCR product sizes will be 625 bp between the yield-positive allele and the non-target allele. The Turkish high-yielding variety Osmancik-97 was used as the donor of *DEP1*. The DEP1-indel1 marker was tested in the donor and 12 recipients, and it discriminated *DEP1* alleles clearly (Fig. 5b). But, when it was applied to intermediate breeding lines, the band intensity of the non-target allele (1031 bp) was markedly lower than that of the positive allele (406 bp) in heterozygotes (plant #2, 3, 5, 6, 7, 10, and 12 in Fig. 5d). To solve the problem of the marker, we developed the tri-primer PCR method, which contained the two primers of DEP1-indel1 marker (DEP1-indel1-F/R) and additional non-target allele-specific primer (DEP1-indel1-625F) (Fig. 5a). As a consequence, the band size of the non-target allele decreased from 1031 bp to 310 bp. The new marker (DEP1-indel1P) worked well in parental lines (Fig. 5c) as well as in heterozygotic plants from intermediate breeding lines (Fig. 5e).

Like our DEP1-indel1 marker, the previously reported *DEP1* marker (Xu et al. 2014) also showed similar results: that the marker was no problem in homozygous plants but the PCR band intensity was quite different between two alleles in heterozygotes. This phenomenon was caused by the difference in PCR amplification efficiency between two different PCR products in the same reaction tube during PCR cycling. Usually, this occurs when the gap between two PCR products is large (several hundred base pairs) such as the DEP1-indel1 marker (625-bp gap) or the indel region contains difficult PCR sequences such as high G-C sequence or inverted repeat sequence. Generally, the longer PCR product and the difficult PCR region show lower amplification efficiency, resulting in a weaker band in agarose gel. To achieve similar PCR efficiency between non-target allele and yield-positive allele, we reduced the gap from 625 bp to 96 bp by adding DEP1-indel1-625F primer (Fig. 5a), resulting in a tri-primer PCR system (DEP1-indel1-F/R/625F). Eventually, the DEP1-indel1P marker generated similar band intensity between non-target allele and positive-allele bands in heterozygotes (Fig. 5e). The marker will be more effective for MAS of the *DEP1* gene than previous marker.

### *SPIKE*/*LSCHL4*/*NAL1*/*GPS* Markers

**Fig. 6:**
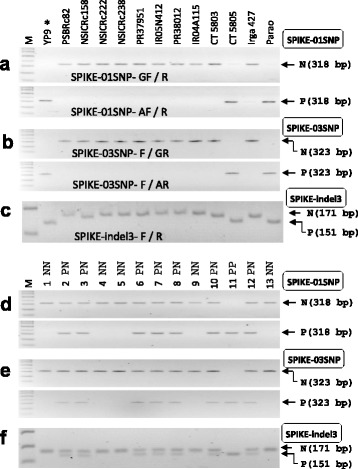
*SPIKE* markers. **a-c** Agarose gel images analyzed by three *SPIKE* markers from the parental lines. The SPIKE-01SNP marker discriminated G/A SNP located on the third exon using two separated PCRs (SPIKE-01SNP- GF/R primer set and SPIKE-01SNP-AF/R primer set) (**a**). The SPIKE-03SNP marker detected the G/A SNP on the fifth exon using two separated PCRs (SPIKE-03SNP-F/GR primer set and SPIKE-03SNP-F/AR primer set) (**b**). The SPIKE-indel3 marker distinguished a 20-bp indel located in the promoter region (**c**). YP9 was used as a donor line of *SPIKE* gene. **d**-**f** Application of the SPIKE-01SNP marker (**d**), the SPIKE-03SNP marker (**e**), and the SPIKE-indel3 marker (**f**) in the intermediate breeding line. Thirteen BC_2_F_1_ plants derived from PSB Rc82 x YP9 cross were genotyped by the three markers, respectively

*SPIKELET NUMBER* (*SPIKE*), *LSCHL4*, and *GPS* are allelic to *Narrow leaf1* (*NAL1*) encoding a plant-specific protein with unknown biochemical function (Qi et al. 2008; Fujita et al. 2013; Takai et al. 2013; Zhang et al. 2014). The *NAL1* gene was originally identified through characterization of a classic rice dwarf mutant, *nal1* (Qi et al. 2008), and the other alleles were characterized by QTLs with map-based cloning by independent research groups. The *SPIKE* allele from *tropical japonica* landrace Daringan and the *LSCHL4* allele from *temperate japonica* variety Nipponbare increased grain number per panicle and grain yield in *indica* backgrounds (Fujita et al. 2013; Zhang et al. 2014). The protein coding sequences of *NAL1* were identical among *japonica* varieties (Daringan, Nipponbare, and Koshikari) and among *indica* varieties (IR64, 93-11, and Takanari), respectively. The *NAL1*-*japonica* allele and the *NAL1*-*indica* allele were distinguished by three SNPs causing amino acid change in CDS (Fujita et al. 2013; Takai et al. 2013; Zhang et al. 2014). Based on the sequence analysis of *NAL1* gene, 16 cultivars were classified into five haplotypes (Takai et al. 2013). Our WGS data showed that Habataki, NSIC Rc222, and NSIC Rc238 had the same DNA sequences as the *NAL1*-Takanari allele (Type 1) (Additional file 4: Figure S4). In the case of NSIC Rc158, several SNPs were newly found but the deduced amino acids were the same as those of the *NAL1*-Takanari allele (Type 1). So, these three recipients may need the *NAL1*- *japonica* allele to increase yield potential. We developed three allele-specific PCR markers: the SPIKE-01SNP marker detecting G/A SNP on the third exon, the SPIKE-03SNP marker detecting G/A SNP on the fifth exon, and the SPIKE-indel3 marker detecting a 20-bp indel in the promoter region (1.9 kb distance from the start codon). These three markers were tested in the donor line and 12 recipients (Fig. 6a-c). Among the 12 recipients, CT5805 and Parao showed the same band pattern as the *SPIKE* donor line (YP9), suggesting that the two varieties had the yield positive *NAL1*- *japonica* allele (Type 5). Therefore, we need to introduce the *NAL1*-Type 5 allele into 10 recipients. The breeding line (BC_2_F_1_) derived from a PSB Rc82 x YP9 cross was analyzed by the three *SPIKE* markers (Fig. 6d-f). As expected, the three markers showed the same results among markers. In conclusion, the SPIKE markers are effective for introducing a Type 5 allele in an *indica* background.

The yield-positive *NAL1* allele originated from *japonica*-type varieties and this allele increased yield in several *indica*-type cultivars (Fujita et al. 2013; Zhang et al. 2014). In the 12 recurrent parents, only two lines (CT5805 and Parao) had the yield-positive *NAL1* allele. This result indicates that the *NAL1*-*japonica* allele will be effective in many *indica* cultivars for increasing yield. Here, we developed three gene-tagged markers, SPIKE-01SNP marker, SPIKE-03SNP marker, and SPIKE-indel3 marker, discriminating the *japonica* allele and *indica* allele. These markers will be useful for MAS of *NAL1* alleles. Through WGS data analysis, we found new two haplotypes (Type 6 and Type 7) to the *NAL1* gene (Additional file 4: Figure S4). Line ST6 producing high grain number per panicle needs to be tested to determine whether it is superior to or has the same allele as the *japonica* allele because an additional amino acid change was found on the fifth exon in ST6.

### *GS5* Markers

**Fig. 7:**
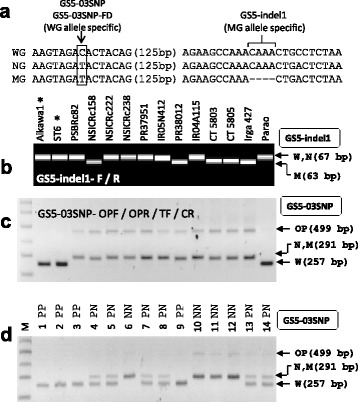
*GS5* markers. **a** The selected DNA polymorphisms in the *GS5* promoter region for marker development. The location of the 4-bp indel is 320-bp distance from the translation initiation codon. The C/T SNP was used for designing GS5-03SNP (PCR-gel-based marker) and GS5-03SNP-FD (Fluidigm genotyping marker) markers. **b** Capillary electrophoresis image from the parental lines analyzed by the GS5-indel1 marker. Aikawa1 and ST6 were used as donors of *GS5*-WG allele. The WG, MG, and NG alleles of the *GS5* gene were designated as W, M, and N, respectively. **c** Agarose gel image analyzed by the GS5-03SNP marker from the parental lines. **d** Application of the GS5-03SNP marker in intermediate breeding line. Fourteen BC_2_F_2_ plants derived from PR37951 x ST6 cross were tested

Abundant transcripts of *GS5*, encoding a putative serine carboxypeptidase, in the hull (palea/lemma) before heading and in the developing endosperm enhanced grain width and grain weight. Through sequence analysis of the *GS5* promoter (2 kb upstream from the start codon) from 35 cultivars, the sequences were divided into three groups: wide grain (WG) allele, medium grain (MG) allele, and narrow grain (NG) allele (Li et al. 2011). First, we checked the *GS5* alleles using WGS data analysis. The sequences of *GS5* of Habataki, ST12, NSIC Rc222, and NSIC Rc238 were the same as those of *GS5*-NG allele. NSIC Rc158 and ST6 belonged to the *GS5*-MG allele and *GS5*-WG allele, respectively. Through PCR product sequencing of *GS5* promoter region, *GS5* alleles were determined from 12 recipients. Parao belonged to the *GS5*-WG allele and NSIC Rc158, PR38012, CT5803, CT5805, and Irga427 belonged to the *GS5*-MG allele. Based on the sequence alignment of *GS5* promoter, we selected the MG-specific 4-bp indel and the WG-specific C/T SNP to design the GS5-indel1 marker and the GS5-03SNP marker, respectively (Fig. 7a). The GS5-indel1 marker produced a 63-bp band (MG allele) and a 67-bp band (WG or NG). The 4-bp difference could not be separated properly in agarose gel. After PCR amplification from the parental lines, the PCR products were analyzed by high-resolution electrophoresis such as PAGE (data not shown) or capillary electrophoresis using Fragment Analyzer (Fig. 7b). The genotyping result was consistent with the DNA sequencing data. To select the WG allele, we developed the GS5-03SNP marker. Genotyping PCR was performed with four primers (GS5-03SNP-OPF/OPR/TF/CR) from the parental lines and electrophoresis was done in agarose gel (Fig. 7c). The genotype result was consistent with sequencing data, indicating that the marker data were reliable. The marker was also validated in intermediate breeding line and discriminated SNPs properly (Fig. 7d).

The *GS5* promoter sequences of our parental lines were classified into the existing three *GS5* alleles (WG, MG, and NG) (Li et al. 2011). In the 12 recurrent parent lines, the numbers of WG, MG, and NG alleles were one, five, and six accessions, respectively. This result indicated that many *indica* varieties had MG or NG alleles. To increase grain size, WG or MG alleles of *GS5* need to be introduced in *indica* cultivars. The GS5-indel1 marker and the GS5-03SNP marker will be helpful for this purpose. For the GS5-03SNP marker, the largest band (499 bp) generated by common outer primers was weaker than the allele-specific bands (257 bp and 291 bp) (Fig. 7c-d). This could be caused by the difference in PCR efficiency between/among bands in one tube. However, this is not a problem because there was no band intensity difference between two allele-specific bands in heterozygotes (Fig. 7d).

### *TGW6* Marker

**Fig. 8:**
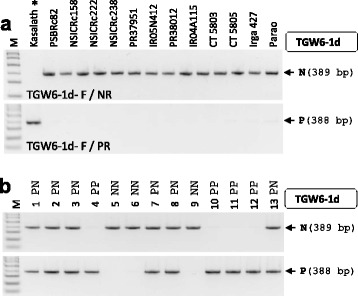
*TGW6* marker. **a** Agarose gel images analyzed by TGW6-1d marker from the donor line (Kasalath) and 12 recipients. The alleles of the *TGW6* gene were determined by TGW6-1d-F/NR primer set detecting the non-target allele and TGW6-1d-F/PR primer set detecting the yield-positive allele. **b** Application of TGW6-1d marker in intermediate breeding line. Thirteen BC_1_F_2_ plants derived from PR38012 x Kasalath cross were tested

*TGW6*, encoding a novel protein having hydrolase activity from IAA-glucose into IAA and glucose, regulates grain weight through controlling both source ability and sink size. *TGW6* is a single-exon gene and the loss-of-function allele caused by a 1-bp deletion in CDS in Kasalath increased grain weight (Ishimaru et al. 2013). We developed the TGW6-1d marker detecting FNP (1-bp deletion) to select a yield-positive allele (*TGW6*-Kasalath allele). The marker consisted of two sets of primers (TGW6-1d-F/NR for the non-target allele and TGW6-1d-F/PR for the yield-positive allele) and it was tested in the donor line (Kasalath) and 12 recipients. All genotypes were determined clearly by this marker and only Kasalath had the yield-positive *TGW6* allele among our breeding materials (Fig. 8a). The marker also functioned properly in intermediate breeding lines (Fig. 8b).

The loss-of-function allele of the *TGW6* gene caused by a 1-bp deletion enhanced grain weight. Among the 69 cultivars, only four varieties had the *TGW6*-Kasalath allele, suggesting that the yield-positive allele is a rare allele (Ishimaru et al. 2013). A similar result was observed in our study. All 12 recipients had the non-yield-positive *TGW6* allele. Therefore, the *TGW6*-Kasalath allele may be effective for increasing grain weight in most cultivars throughout *indica-* and *japonica*-type varieties. The TGW6-1d marker detecting the FNP will be useful for introducing the *TGW6*-Kasalath allele through MAS.

### High-throughput Genotyping Markers Using Fluidigm SNP Genotyping Platform

**Fig. 9:**
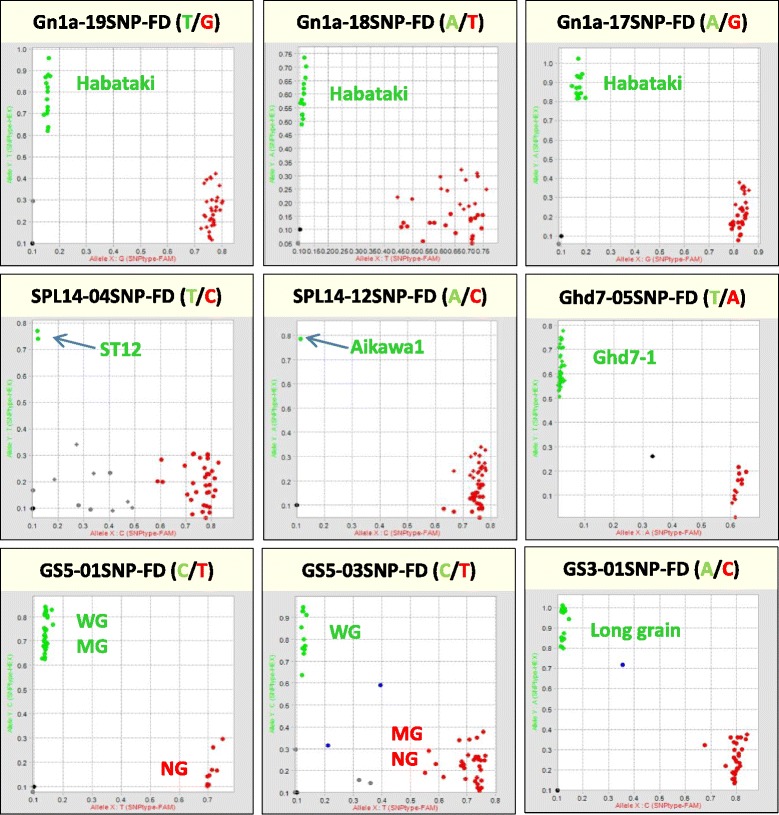
Genotyping of five yield-related genes from 39 rice accessions using nine Fluidigm SNP genotyping markers. The marker name with the detected-nucleotide polymorphism was shown on the top of each graph. The allele-specific primers were labeled with HEX fluorescence dye (*green*) for the yield-positive allele and with FAM fluorescence dye (*red*) for the non-target allele. After PCR on Fluidigm array, the alleles of samples were determined by fluorescent signal intensity. In each marker result, the yield-positive allele of the genes was shown with a green letter. Black spot, non-template control; gray spot, invalid call; blue spot, heterozygote

Currently, several high-throughput genotyping platforms using a fluorescence detection system are available in rice (Thomson 2014). We also developed fluorescence-labeled allele-specific markers using Fluidigm SNP genotyping platform for high-throughput MAS of yield-related genes. For the *Gn1a* gene, three SNPs in the promoter region (Fig. 1a) were discriminated by Gn1a-19SNP-FD, Gn1a-18SNP-FD, and Gn1a-17SNP-FD markers (Fig. 9). For the *OsSPL14* gene, the ST12-specific SNP in the promoter region and the Aikawa1-specific SNP on the third exon (Fig. 2a) were detected by SPL14-04SNP-FD marker and SPL14-12SNP-FD marker, respectively. In the *Ghd7* gene, the 521st nucleotide (A/T) in CDS was used for developing the Ghd7-05SNP-FD marker. We developed the GS5-01SNP-FD marker distinguishing T (NG allele)/C (MG or WG allele) SNP in the *GS5* promoter region (1594-nucleotide upstream from the start codon of H94 line, GenBank accession no. JN256057). And, for selection of the *GS5*-WG allele, the GS5-03SNP-FD marker was developed. For the *GS3* gene, FNP (C/A SNP) on the second exon was used for developing the GS3-01SNP-FD marker. All markers were tested in 47 samples consisting of 39 accessions (Fig.9; Additional file 5: Table S1). Among these, ST12 and Aikawa1 were unique at the SPL14-04SNP-FD marker and SPL14-12SNP-FD marker, respectively (Fig. 9). The genotyping results were consistent with the DNA sequencing data as well as the PCR-gel-based allele-specific markers developed in this study.

In marker-assisted breeding, breeders sometimes handle several hundred samples and/or many markers. Usually, MAS should be finished before flowering to make a backcross or a cross between different gene sources for gene pyramiding. In this case, high-throughput genotyping systems are very helpful. Here, we employed Fluidigm SNP genotyping system. In this system, 24-192 samples can be analyzed with 24-96 customized markers simultaneously. After PCR, fluorescence from the allele-specific PCR products can be detected without gel electrophoresis, and data calling can be computed rapidly. We developed nine Fluidigm SNP markers for five yield-related genes. These markers will be useful for high-throughput genotyping of yield-related genes in rice.

## Conclusion

Here, we described the procedures of SNP/indel marker development for yield-enhancing genes, including allele determination of target genes in the recurrent parental lines, DNA polymorphism screening using WGS and Sanger sequencing techniques, the selection of DNA polymorphisms for marker designing, and marker validation. This procedure can be applied to designing markers for other agronomic traits. For rapid improvement of genetic yield potential in rice, we need to evaluate and use the previously identified yield-enhancing genes in local favorite cultivar backgrounds. The allele-specific markers for yield-enhancing genes shown in this study will be very helpful for MAS of yield-enhancing traits/genes. Through whole-genome sequencing, we identified some new haplotypes for some yield-enhancing genes such as the *SCM2*-ST6 allele and *SPIKE*-ST6 allele. For the same gene, the alleles superior to the previously identified alleles can be found. Therefore, if the new allele was found in an advanced phenotype line such as ST6, the effect of the new allele needs to be tested. To increase rice productivity, the identification of new yield-enhancing genes, isolation of superior alleles of previously identified genes, marker development of the genes for MAS, evaluation of the effect of the genes/alleles, and their use in actual breeding programs should be performed rapidly and updated continuously.

## Methods

### Plant Materials

Habataki, ST12, ST6, Aikawa1, YP9, Osmancik-97, and Kasalath varieties were used as donors of yield-positive alleles of yield-related genes. In addition, 12 elite *indica* varieties originating from the International Rice Research Institute (IRRI), Philippines, and International Center for Tropical Agriculture (CIAT), Colombia, were used as recipients to increase genetic yield potential through the introduction or pyramiding of yield-enhancing genes (Table 1). Seeds of the donor lines Habataki, ST12, ST6, Aikawa1, and Kasalath were obtained from the Bioscience and Biotechnology Center, Nagoya University, Japan, and seeds of Osmancik-97 were obtained from Trakya Agricultural Research Institute, Edirne, Turkey. The plants were grown in the IRRI paddy field, Laguna, Philippines, in 2012-2015. To introduce the yield-positive alleles of yield-related genes, crosses were made between the donor lines and recipients followed by selfing, backcrossing, and crossing with other cross combinations in the same background, based on the breeding plan. The markers we developed were applied to intermediate breeding lines for MAS.

### Whole-genome Sequencing and Comparative Sequence Analysis

In order to identify nucleotide polymorphisms of yield-enhancing genes between the donors and recipients, WGS was performed using Illumina HiSeq 2000 by the Yale Genomics Center (Connecticut, USA) for three donors (Habataki, ST6, and ST12) and three recipients (NSIC Rc158, NSIC Rc222, and NSIC Rc238). The sequencing results yielded 609,301,492 reads in all. Total mapped reads ranged from 47.65 % to 57.69 % and from 50.29 % to 58.42 % against Nipponbare and 93-11 genomes, respectively. Uniquely mapped reads were on average 42.01 % (Additional file 6: Table S2). The short reads were aligned against the Nipponbare genome (MSU v7). The SNP and indel polymorphisms were screened using IGV software (Robinson et al. 2011; https://www.broadinstitute.org/igv/).

### PCR Product Sequencing

To confirm DNA polymorphisms obtained by WGS and to determine the alleles of yield-related genes from 12 recipients, the PCR products containing putative FNPs or donor-specific polymorphisms for yield-related genes were sequenced by the Sanger method. Genomic DNAs were extracted from 12 recipients and four donors (Habataki, ST12, ST6, and Aikawa1) using the CTAB method (Kim et al. 2011) and PCR amplification was performed for *Gn1a*, *OsSPL14*, *SCM2*, *Ghd7*, and *GS5* genes. The primers for preparation of PCR product and DNA sequencing are listed in Additional file 7: Table S3. DNA sequencing reactions were performed with BigDye terminator sequencing kit v3.1 (Applied Biosystems, www.appliedbiosystems.com/), and then the sequences were determined by Applied Biosystems 3730xl DNA analyzer by Macrogen Inc., Republic of Korea (www.macrogen.com/).

### Development of Allele-specific Markers

The DNA sequences of yield-related genes were obtained from NCBI GenBank (www.ncbi.nlm.nih.gov/) using the accession codes presented in the original papers (Ashikari et al. 2005; Ikeda et al. 2007; Xue et al. 2008; Huang et al. 2009; Miura et al. 2010; Li et al. 2011; Ishimaru et al. 2013; Takai et al. 2013). To find DNA polymorphsims between the donors and recipients, multiple sequence alignments were performed using BioEdit software (Hall 1999). Special DNA variations such as FNP, putative FNP, donor-specific polymorphisms, and polymorphisms between the donors and recipients were selected for marker designing. For indel variation, the forward and reverse primers covered the indel region. When the gap was small (less than 20 bp), we reduced PCR product size (<120 bp) to increase the degree of band separation in agarose gel. To discriminate SNP-type DNA polymorphisms, first we used the tetra-primer PCR method (Ye et al. 2001). This type of marker can reveal a SNP by one PCR reaction with four primers. The Gn1a-17 SNP marker and GS5-03 SNP marker were developed based on this method. Secondly the separated allele-specific PCR method was used to design the other markers. This method requires two separate PCR reactions with allele-1-specific primer and allele-2-specific primer, respectively, to define the SNP. Actually, we tried the tetra-primer PCR method for all SNP polymorphisms because of its convenience, but the results from some testing markers were unstable among trials. So, we accepted the separated allele-specific PCR method. To increase allele-specific primer annealing, an artificial-mismatched nucleotide was given near the 3’ end of the allele-specific primer (Hayashi et al. 2004). For better understanding, the designing of the Gn1a-17 SNP marker and the SPIKE-01 SNP marker are shown in Additional file 2: Figure S2. The primer information for allele-specific markers is presented in Table 2.

**Table 2:** Primer information of the allele-specific markers for eight yield-enhancing genes

Marker	DNA polymorphism	Primer	Primer sequence (5' to 3')^a^
Gn1a-17SNP	G/A SNP in promoter	Gn1a-17SNP-OPF	TCGCAGGCACTGCACTTCA
		Gn1a-17SNP-OPR	GCCACCCTAGGTTTGATTCC
		Gn1a-17SNP-AF	CATACCTAGCGTTCTATGCtGA
		Gn1a-17SNP-GR	GGAAGATAAAGAAATTTCACATaCC
Gn1a-indel3	~70-bp indel near the 3'UTR	Gn1a-indel3-F	GATCTAGATGCTCCAAAGTCC
		Gn1a-indel3-R	CTGTACGTACGTGCACGTAG
Gn1a-indel1	16-bp indel in the 5'UTR	Gn1a-indel1-F	GCCACCTTGTCCCTTCTACA
		Gn1a-indel1-R	TGCCATCCTGACCTGCTCT
SPL14-04SNP	C/T SNP in promoter	SPL14-04SNP-F	TAGCCATAGCTTCTGCGTGA
		SPL14-04SNP-CR	ACCGTGCTTACCGCCtGG
		SPL14-04SNP-TR	ACCGTGCTTACCGCCtGA
SPL14-12SNP	C/A SNP on the third exon	SPL14-12SNP-R	CAAGTGAGACTTCATGTGGT
		SPL14-12SNP-CF	ACCGACTCGAGCTGTGtTC
		SPL14-12SNP-F	GTTCAGAAGCTTTACGTTGGA
		SPL14-12SNP-AR	GCTGGGTTGACAGAAGAGAtAT
SCM2-indel1	12-bp indel in promoter	SCM2-indel1-F	GGAAATGATGAACACTGTCCA
		SCM2-indel1-R	GTTTGTCTCAGCTCTGATCTG
Ghd7-05SNP	A/T SNP on the second exon	Ghd7-05SNP-F	TGCTTATGCGTACATCTGGAT
		Ghd7-05SNP-AR	TGGGTTCAAGCTCTCCaCAT
		Ghd7-05SNP-TR	TGGGTTCAAGCTCTCCaCAA
DEP1-indel1P		DEP1-indel1-F	GCAAGTGCTCACCCAAGTG
	625-bp indel on the fifth exon	DEP1-indel1-R	GTTCGAACTTAATCAAAGGCCT
		DEP1-indel1-625F	CACGACGCAGTGCTTCAGCT
SPIKE-01SNP	G/A SNP on the third exon	SPIKE-01SNP-GF	GGTTGGTTTCCTCACTAAaCG
		SPIKE-01SNP-AF	GGTTGGTTTCCTCACTAAaCA
		SPIKE-01SNP-R	ATGGGAACTAGGAAGCAGGA
SPIKE-03SNP	G/A SNP on the fifth exon	SPIKE-03SNP-F	CTACTCGACCGTCTGGAAC
		SPIKE-03SNP-GR	TGGCTCGAAGATCTCTTCtAC
		SPIKE-03SNP-AR	TGGCTCGAAGATCTCTTCtAT
SPIKE-indel3	~20-bp indel in promoter	SPIKE-indel3-F	GGAGAGACATGGACGGCT
		SPIKE-indel3-R	TGGTGGCGATCATGCTGC
GS5-indel1	4-bp indel in promoter	GS5-indel1-F	CTAACTCCCATGGAATTACTAG
		GS5-indel1-R	GGAAAGCGAAACTGATTGACA
GS5-03SNP	T/C SNP in promoter	GS5-03SNP-OPF	ACTTTCAACTAAAGTGATATTACCTC
		GS5-03SNP-OPR	TCTATATATCCATCGTCCATGGTG
		GS5-03SNP-TF	CGCAGCCTAACTACCTAAGTAGcT
		GS5-03SNP-CR	ACATGCGTGCCAATATTCCTGTAtTG
TGW6-1d	1-bp indel on the first exon	TGW6-1d-F	GCCAACTGATCAGACTGAG
		TGW6-1d-NR	CGTGGGGAGAGTCGATtCC
		TGW6-1d-PR	CGTGGGGAGAGTCGATtCG

^a^Lower case nucleotide near the 3' end of allele-specific primer represents the artificial mismatched nucleotide for increasing allele specificity during primer annealing step of PCR.

### DNA Preparation, PCR with Allele-specific Markers, and Electrophoresis

Leaf samples were collected from the intermediate breeding lines in the paddy field. Fresh or stored leaf (-20 °C) was cut at about 4 cm long and was placed into a 2-mL tube with a steel ball. Then, the tubes were frozen in liquid nitrogen and leaf tissues were ground with the 2010 Geno/Grinder (www.spexsampleprep.com). In each tube, 200 μL of DNA extraction buffer (100 mM Tris-HCl, pH 9.5, 1 M KCl, 10 mM EDTA, pH 8.0) was added and the samples were incubated at 65°C for 30 min. The samples were diluted by adding 1 mL of water and centrifuged for 10 min at the maximum speed. The supernatant was transferred into a 96-well plate and stored at 4 °C for further uses. All genotyping markers followed the PCR conditions mentioned below. The 20-μL PCR solution contained 1× PCR buffer, 200 μM of each dNTP, 0.25 μM of each primer, 1.5 μL of supernatant (template DNA), and 1 unit of *Taq* DNA polymerase. Thermal cycles were programmed as follows: 94 °C, 3 min; 35 cycles of 95°C for 25 s; 55 °C for 25 s; and 72 °C for 35 s, concluding with 72 °C for 5 min. The PCR products amplified with Gn1a-indel1 marker, SCM2-indel1 marker, and SPIKE-indel3 marker were electrophoresed in 4 % agarose gel and the others in 2.5 % agarose gel. In the case of GS5-indel1 marker, its PCR products (63 bp/67 bp) could not be separated properly in agarose gel. So, it was analyzed by 8 % polyacrylamide gel electrophoresis (PAGE) or capillary electrophoresis.

### High-throughput Capillary Electrophoresis

Gel electrophoresis requires some labor and time because of manual gel preparation and hand loading of PCR products. Currently, automated high-throughput capillary electrophoresis instruments are available and are helpful for handling a huge number of samples. Here, we used Fragment Analyzer (Advanced Analytical Technologies Inc., www.aati-us.com/) that supports ready-to-use polymer (gel), automated sample loading, and data calling. The PCR products produced by the GS5-indel1 marker and the SCM2-indel1 marker were analyzed by Fragment Analyzer.

### Fluidigm SNP Genotyping

We selected nine SNPs from five yield-related genes to design Fluidigm SNP genotyping markers. The sequences, including both 250-bp upstream and 250-bp downstream of the target SNP, were sent to Fluidigm (Assay_Design_Group@fluidigm.com) and the Fluidigm SNP genotyping markers consisting of specific target amplification (STA) primer, locus-specific (LS) primer, and two allele-specific primers were designed (Additional file 8: Table S4). Forty-seven genomic DNAs were prepared from 39 rice accessions using the CTAB method (Kim et al. 2011). Genotyping was performed following the Fluidigm SNP genotyping manual by the IRRI genotyping service laboratory (gsl@irri.org). Briefly, the target region was amplified with STA primer and LS primer under a thermal cycler. The diluted PCR products from 47 samples, nine Fluidigm SNP markers, and PCR reagents were simultaneously mated in FR48.48 Dynamic array by IFC Controller. Then, PCR was performed in FC1™ Cycler, and finally the fluorescence signals from the end PCR products were read under EP1^TM^ Reader.

### Statement

I confirm that we have followed the guide lines of the government of Philippines and the policies of IRRI for growing rice plants and carrying out research for this study.

## Additional files

Additional file 1: Figure S1. Screening of DNA polymorphisms in the *Gn1a* gene using WGS data. Sequencing reads were aligned to the reference genome sequence (IRGSP-1.0). Note that the *Gn1a* gene lay on the opposite strand of the reference sequence. Screen-captured images of IGV software showed nucleotide variations that were used for marker development. (**A**) Three SNPs located in the *Gn1a* promoter region were shown with their genomic location on chromosome 1. Chr1: 5276405, Chr1: 5276521, and Chr1: 5276591 SNPs were used for Gn1a-17SNP/Gn1a-17SNP-FD, Gn1a-18SNP-FD, and Gn1a-19SNP-FD markers, respectively. (**B**) About a 70-bp deletion (red circles) near the 3’ UTR of *Gn1a* was found in varieties NSIC Rc158 and NSIC Rc238. This indel was used for designing the Gn1a-indel3 marker. (DOC 178 kb) Additional file 2: Figure S2. Marker designing schemes for SNP-type polymorphisms. (**A**) Schematic presentation of the tetra-primer PCR method for designing Gn1a-17SNP marker. The target SNPs, G and A, are highlighted in each genome and its surrounding sequences are represented with the allele-specific primers (pink, G allele-specific primer; green, A allele-specific primer). Actually, the SNP is determined by the last nucleotide (filled triangle) of the allele-specific primer. Proper annealing of the last nucleotide of the primer (the 3’ end) is very important for PCR amplification because *Taq* DNA polymerase start polymerization at that nucleotide through adding dNTP. For instance, the A allele-specific primer (green) can be annealed to the G allele genome but the efficiency of DNA polymerization will be very low because of no annealing of the 3’ end of the primer, resulting in no PCR band or a very weak band. To increase allele specificity, we gave an artificial mismatched nucleotide near the 3’ end (second or third nucleotide from the 3’ end) of the allele-specific primer (lowercase with underlined nucleotide in Figure). Primer combination of the Gn1a-17SNP marker, its predicted band size, and deduced gel image depending on genotypes were presented. (**B**) Schematic presentation of the separated allele-specific PCR method for designing the SPIKE-01SNP marker. To discriminate G/A SNP, the SPIKE-01SNP marker consisted of two PCRs (PCR #1, SPIKE-01SNP-GF/R; PCR #2, SPIKE-01SNP-AF/R). Between the G allele-specific primer (pink) and A allele-specific primer (green), only the last nucleotide of each allele-specific primer (filled triangle) is different. To increase allele specificity, the artificial mismatched nucleotide near the 3’ end was given in the allele-specific primer. PCRs were performed with each allele-specific primer and common reverse primer. Primer combinations of the SPIKE-01SNP marker, its predicted band size, and deduced gel image depending on genotypes were presented. (**C**) The effect of artificial mismatched nucleotide near the 3’ end of the allele-specific primer. As an example, the SPIKE-01SNP marker was designed without the artificial mismatched nucleotide in allele-specific primers that were shown on the gel image. Non-allele-specific PCR amplifications were obtained with these primers. Using artificial mismatch, we obtained allele-specific PCR amplifications (Fig. 6a). (DOC 240 kb) Additional file 3: Figure S3. Screening of ST12-specific DNA variation in the *OsSPL14* promoter region through WGS data analysis. Note that the *OsSPL14* gene lay on the opposite strand of the reference sequence. The reference genome sequence was shown at the bottom of the image. Screen-captured image of IGV software showed an ST12-specific SNP located at the Chr 8: 25282790 nucleotide position (IRGSP-1.0). (DOC 77 kb) Additional file 4: Figure S4. Haplotype analysis of the *NAL1* gene from six varieties using WGS data according to the previously analyzed *NAL1* haplotypes (Takai et al. 2013). The nucleotide position was calculated based on the previous report (1 = the starting nucleotide of 5’ UTR in Takanari) and newly identified polymorphisms in this study were highlighted by red characters. The 1640-position nucleotide (G/A) and the 2884-position nucleotide (G/A) were used for designing the SPIKE-01SNP marker and SPIKE-03 SNP marker, respectively. S.S., synonymous nucleotide substitution. (DOC 78 kb) Additional file 5: Table S1. Summary of Fluidigm SNP genotyping results. (DOC 90 kb) Additional file 6: Table S2. Summary of whole-genome sequencing of six varieties. (DOC 47 kb) Additional file 7: Table S3. Primers for preparation of PCR products and PCR product sequencing. (DOC 35 kb) Additional file 8: Table S4. Markers for Fluidigm SNP genotyping platform. (DOC 36 kb)

## Abbreviations

MAS: Marker-assisted selection
FNP: Functional nucleotide polymorphism
QTL: Quantitative trait loci
CDS: Coding DNA sequence
CAPS: Cleaved amplified polymorphic sequence
SNP: Single nucleotide polymorphism
WGS: Whole-genome sequencing
indel: Insertion/deletion
NIL: Near-isogenic line
