# Supplementary material for: Development and validation of allele-specific SNP/indel markers for eight yield-enhancing genes using whole-genome sequencing strategy to increase yield potential of rice, Oryza sativa L
Source: Rice (N Y). 2016 Mar 18;9:12. doi: 10.1186/s12284-016-0084-7 (PMC4797370; doi:10.1186/s12284-016-0084-7)
Supplement: Additional file 1: Figure S1. — Screening of DNA polymorphisms in the Gn1a gene using WGS data. Sequencing reads were aligned to the reference genome sequence (IRGSP-1.0). Note that the Gn1a gene lay on the opposite strand of the reference sequence. Screen-captured images of IGV software showed nucleotide variations that were used for marker development. (A) Three SNPs located in the Gn1a promoter region were shown with their genomic location on chromosome 1. Chr1: 5276405, Chr1: 5276521, and Chr1: 5276591 SNPs were used for Gn1a-17SNP/Gn1a-17SNP-FD, Gn1a-18SNP-FD, and Gn1a-19SNP-FD markers, respectively. (B) About a 70-bp deletion (red circles) near the 3’ UTR of Gn1a was found in varieties NSIC Rc158 and NSIC Rc238. This indel was used for designing the Gn1a-indel3 marker. (DOC 178 kb) [file 12284_2016_84_MOESM1_ESM.doc]

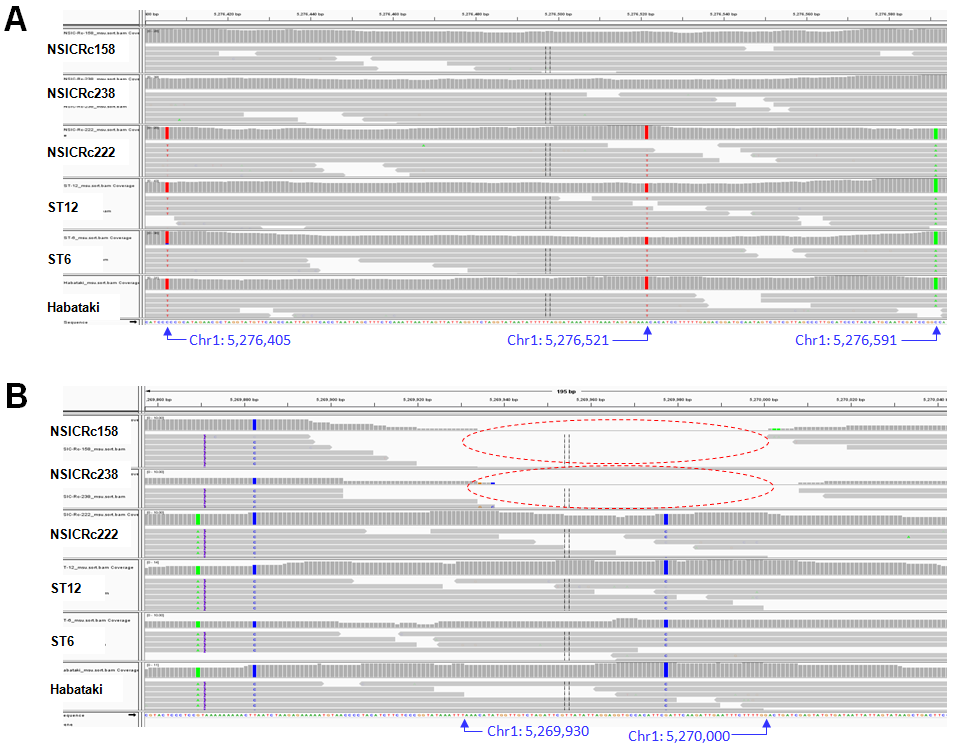


**Additional file 1: Figure S1** **Screening of DNA polymorphisms in the *Gn1a* gene using WGS data.** Sequencing reads were aligned to the reference genome sequence (IRGSP-1.0). Note that the *Gn1a* gene lay on the opposite strand of the reference sequence. Screen-captured images of IGV software showed nucleotide variations that were used for marker development. (**A**) Three SNPs located in the *Gn1a* promoter region were shown with their genomic location on chromosome 1. Chr1: 5276405, Chr1: 5276521, and Chr1: 5276591 SNPs were used for Gn1a-17SNP/Gn1a-17SNP-FD, Gn1a-18SNP-FD, and Gn1a-19SNP-FD markers, respectively. (**B**) About a 70-bp deletion (red circles) near the 3’ UTR of *Gn1a* was found in varieties NSIC Rc158 and NSIC Rc238. This indel was used for designing the Gn1a-indel3 marker.
