# Supplementary material for: Development and validation of allele-specific SNP/indel markers for eight yield-enhancing genes using whole-genome sequencing strategy to increase yield potential of rice, Oryza sativa L
Source: Rice (N Y). 2016 Mar 18;9:12. doi: 10.1186/s12284-016-0084-7 (PMC4797370; doi:10.1186/s12284-016-0084-7)
Supplement: Additional file 2: Figure S2. — Marker designing schemes for SNP-type polymorphisms. (A) Schematic presentation of the tetra-primer PCR method for designing Gn1a-17SNP marker. The target SNPs, G and A, are highlighted in each genome and its surrounding sequences are represented with the allele-specific primers (pink, G allele-specific primer; green, A allele-specific primer). Actually, the SNP is determined by the last nucleotide (filled triangle) of the allele-specific primer. Proper annealing of the last nucleotide of the primer (the 3’ end) is very important for PCR amplification because Taq DNA polymerase start polymerization at that nucleotide through adding dNTP. For instance, the A allele-specific primer (green) can be annealed to the G allele genome but the efficiency of DNA polymerization will be very low because of no annealing of the 3’ end of the primer, resulting in no PCR band or a very weak band. To increase allele specificity, we gave an artificial mismatched nucleotide near the 3’ end (second or third nucleotide from the 3’ end) of the allele-specific primer (lowercase with underlined nucleotide in Figure). Primer combination of the Gn1a-17SNP marker, its predicted band size, and deduced gel image depending on genotypes were presented. (B) Schematic presentation of the separated allele-specific PCR method for designing the SPIKE-01SNP marker. To discriminate G/A SNP, the SPIKE-01SNP marker consisted of two PCRs (PCR #1, SPIKE-01SNP-GF/R; PCR #2, SPIKE-01SNP-AF/R). Between the G allele-specific primer (pink) and A allele-specific primer (green), only the last nucleotide of each allele-specific primer (filled triangle) is different. To increase allele specificity, the artificial mismatched nucleotide near the 3’ end was given in the allele-specific primer. PCRs were performed with each allele-specific primer and common reverse primer. Primer combinations of the SPIKE-01SNP marker, its predicted band size, and deduced gel image depending on genotypes were presented. (C) The effect of [file 12284_2016_84_MOESM2_ESM.doc]

**
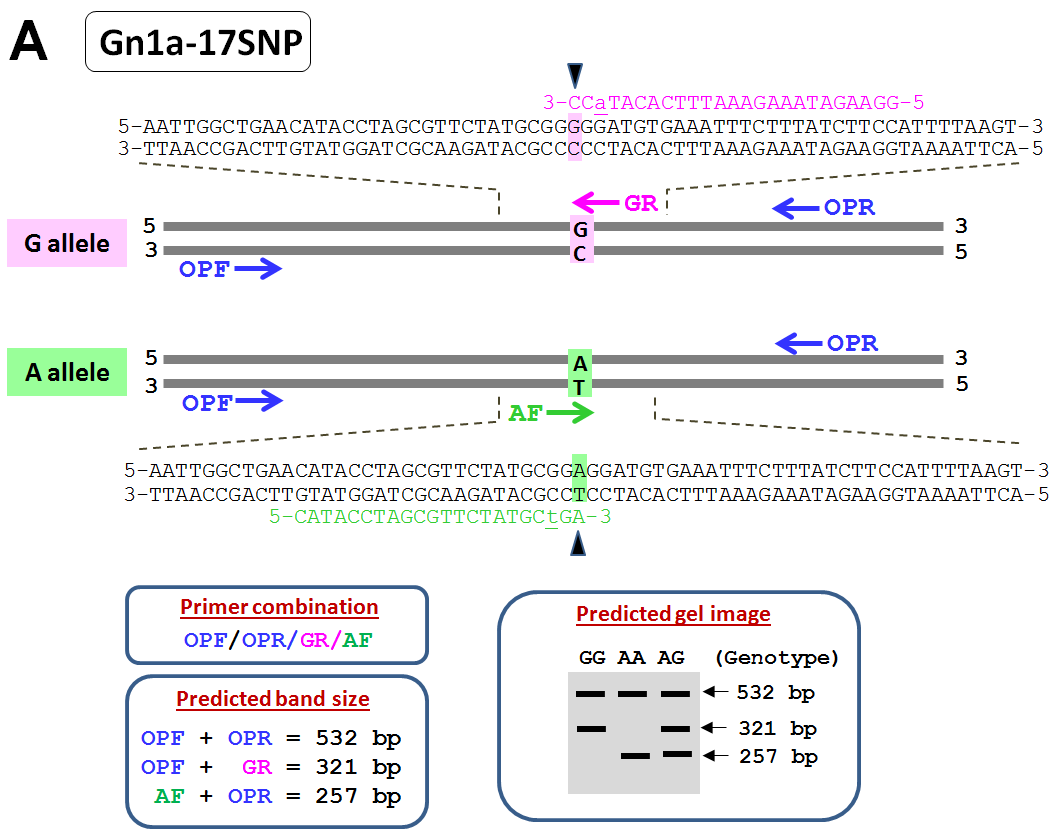
**

**
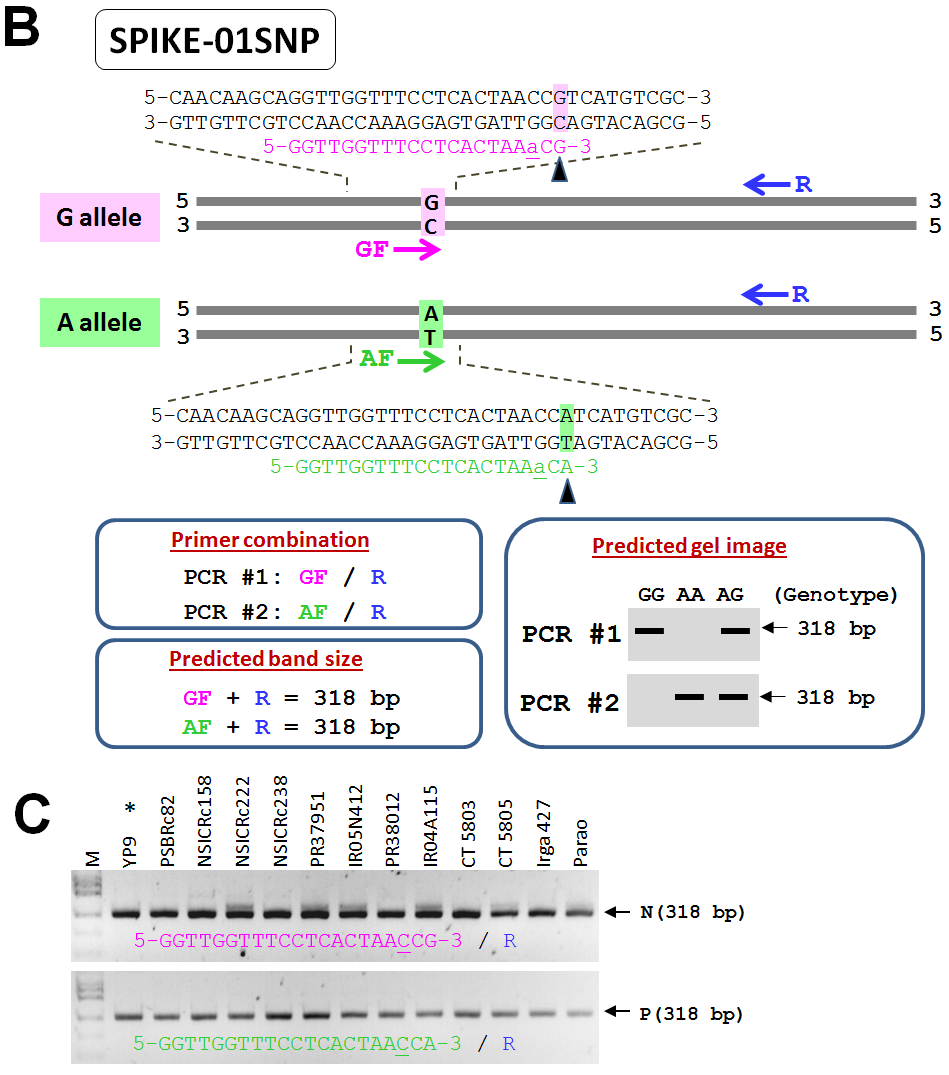
**

**Additional file 2: Figure S2 Marker designing schemes for SNP-type polymorphisms.** (**A**) Schematic presentation of the tetra-primer PCR method for designing Gn1a-17SNP marker. The target SNPs, G and A, are highlighted in each genome and its surrounding sequences are represented with the allele-specific primers (pink, G allele-specific primer; green, A allele-specific primer). Actually, the SNP is determined by the last nucleotide (filled triangle) of the allele-specific primer. Proper annealing of the last nucleotide of the primer (the 3’ end) is very important for PCR amplification because *Taq* DNA polymerase start polymerization at that nucleotide through adding dNTP. For instance, the A allele-specific primer (green) can be annealed to the G allele genome but the efficiency of DNA polymerization will be very low because of no annealing of the 3’ end of the primer, resulting in no PCR band or a very weak band. To increase allele specificity, we gave an artificial mismatched nucleotide near the 3’ end (second or third nucleotide from the 3’ end) of the allele-specific primer (lowercase with underlined nucleotide in Figure). Primer combination of the Gn1a-17SNP marker, its predicted band size, and deduced gel image depending on genotypes were presented. (**B**) Schematic presentation of the separated allele-specific PCR method for designing the SPIKE-01SNP marker. To discriminate G/A SNP, the SPIKE-01SNP marker consisted of two PCRs (PCR #1, SPIKE-01SNP-GF/R; PCR #2, SPIKE-01SNP-AF/R). Between the G allele-specific primer (pink) and A allele-specific primer (green), only the last nucleotide of each allele-specific primer (filled triangle) is different. To increase allele specificity, the artificial mismatched nucleotide near the 3’ end was given in the allele-specific primer. PCRs were performed with each allele-specific primer and common reverse primer. Primer combinations of the SPIKE-01SNP marker, its predicted band size, and deduced gel image depending on genotypes were presented. (**C**) The effect of artificial mismatched nucleotide near the 3’ end of the allele-specific primer. As an example, the SPIKE-01SNP marker was designed without the artificial mismatched nucleotide in allele-specific primers that were shown on the gel image. Non-allele-specific PCR amplifications were obtained with these primers. Using artificial mismatch, we obtained allele-specific PCR amplifications (Fig. 6a).
