# Supplementary material for: Development and validation of allele-specific SNP/indel markers for eight yield-enhancing genes using whole-genome sequencing strategy to increase yield potential of rice, Oryza sativa L
Source: Rice (N Y). 2016 Mar 18;9:12. doi: 10.1186/s12284-016-0084-7 (PMC4797370; doi:10.1186/s12284-016-0084-7)
Supplement: Additional file 3: Figure S3. — Screening of ST12-specific DNA variation in the OsSPL14 promoter region through WGS data analysis. Note that the OsSPL14 gene lay on the opposite strand of the reference sequence. The reference genome sequence was shown at the bottom of the image. Screen-captured image of IGV software showed an ST12-specific SNP located at the Chr 8: 25282790 nucleotide position (IRGSP-1.0). (DOC 77 kb) [file 12284_2016_84_MOESM3_ESM.doc]

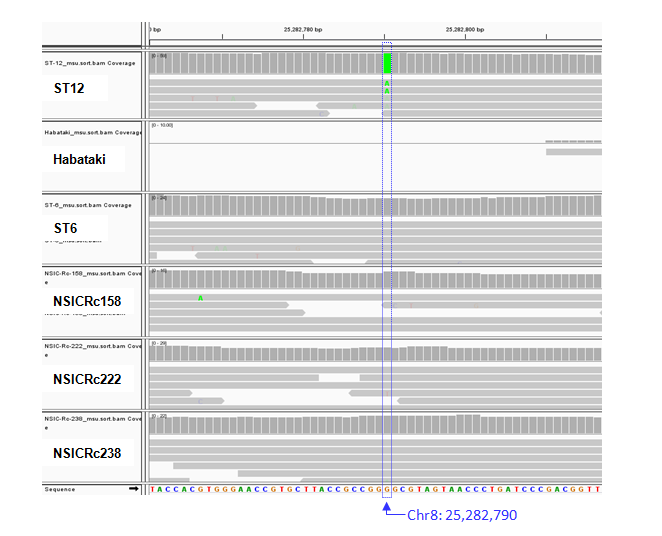


**Additional file 3: Figure S3** **Screening of ST12-specific DNA variation in the *OsSPL14* promoter region through WGS data analysis.** Note that the *OsSPL14* gene lay on the opposite strand of the reference sequence. The reference genome sequence was shown at the bottom of the image. Screen-captured image of IGV software showed an ST12-specific SNP located at the Chr 8: 25282790 nucleotide position (IRGSP-1.0).
