# Supplementary material for: Development and validation of allele-specific SNP/indel markers for eight yield-enhancing genes using whole-genome sequencing strategy to increase yield potential of rice, Oryza sativa L
Source: Rice (N Y). 2016 Mar 18;9:12. doi: 10.1186/s12284-016-0084-7 (PMC4797370; doi:10.1186/s12284-016-0084-7)
Supplement: Additional file 4: Figure S4. — Haplotype analysis of the NAL1 gene from six varieties using WGS data according to the previously analyzed NAL1 haplotypes (Takai et al. 2013). The nucleotide position was calculated based on the previous report (1 = the starting nucleotide of 5’ UTR in Takanari) and newly identified polymorphisms in this study were highlighted by red characters. The 1640-position nucleotide (G/A) and the 2884-position nucleotide (G/A) were used for designing the SPIKE-01SNP marker and SPIKE-03 SNP marker, respectively. S.S., synonymous nucleotide substitution. (DOC 78 kb) [file 12284_2016_84_MOESM4_ESM.doc]

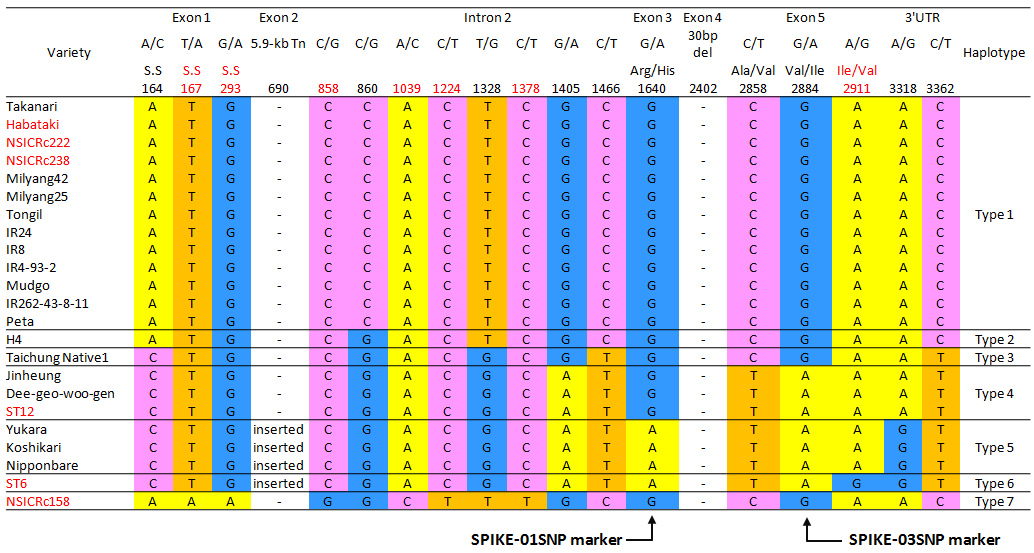


**Additional file 4: Figure S4** **Haplotype analysis of the *NAL1* gene from six varieties using WGS data according to the previously analyzed *NAL1* haplotypes (Takai et al. 2013).** The nucleotide position was calculated based on the previous report (1 = the starting nucleotide of 5’ UTR in Takanari) and newly identified polymorphisms in this study were highlighted by red characters. The 1640-position nucleotide (G/A) and the 2884-position nucleotide (G/A) were used for designing the SPIKE-01SNP marker and SPIKE-03 SNP marker, respectively. S.S., synonymous nucleotide substitution.
