# Supplementary material for: Development and validation of allele-specific SNP/indel markers for eight yield-enhancing genes using whole-genome sequencing strategy to increase yield potential of rice, Oryza sativa L
Source: Rice (N Y). 2016 Mar 18;9:12. doi: 10.1186/s12284-016-0084-7 (PMC4797370; doi:10.1186/s12284-016-0084-7)
Supplement: Additional file 5: Table S1. — Summary of Fluidigm SNP genotyping results. (DOC 90 kb) [file 12284_2016_84_MOESM5_ESM.doc]

**Additional file 5: Table S1.** **Summary of Fluidigm SNP genotyping results**

| No. | Sample^a^ | Type^b^ | Gn1a-19SNP-FD | Gn1a-18SNP-FD | Gn1a-17SNP-FD | SPL14-04SNP-FD | SPL14-12SNP-FD | Ghd7-05SNP-FD | GS5-01SNP-FD | GS5-03SNP-FD | GS3-01SNP-FD |
| --- | --- | --- | --- | --- | --- | --- | --- | --- | --- | --- | --- |
| 1 | *O. rufipogon* 104423 | WS | GG | TT | GG | Invalid | CC | TT | CC | CC | CC |
| 2 | *O. rufipogon* 105491 | WS | TT | AA | AA | Invalid | CC | TT | CC | CC | CC |
| 3 | Aikawa1 | ja | GG | TT | GG | CC | AA | AA | CC | CC | CC |
| 4 | Asominori (p1) | ja | GG | TT | GG | CC | CC | AA | CC | CC | CC |
| 5 | Asominori (p2) | ja | GG | TT | GG | CC | CC | AA | CC | CC | CC |
| 6 | Aswin | in | TT | AA | AA | CC | CC | TT | CC | TT | CC |
| 7 | Azucena | LA | GG | TT | GG | CC | CC | AA | CC | No call | AA |
| 8 | Chuan7 | in | TT | AA | AA | CC | CC | TT | CC | CC | CC |
| 9 | CT5803 | in | GG | TT | GG | CC | CC | TT | CC | TT | AA |
| 10 | CT5805 | in | TT | AA | AA | CC | CC | TT | CC | TT | AA |
| 11 | FR13a | LA | TT | AA | AA | Invalid | CC | TT | CC | CC | CC |
| 12 | H94 | in | GG | TT | GG | CC | CC | TT | TT | TT | CC |
| 13 | Habataki (p1) | in | TT | AA | AA | Invalid | CC | TT | TT | TT | CC |
| 14 | Habataki (p2) | in | TT | AA | AA | Invalid | CC | TT | TT | TT | CC |
| 15 | Halilbey | ja | GG | TT | GG | CC | CC | AA | CC | No call | AA |
| 16 | IR24-P | in | TT | AA | AA | CC | CC | TT | CC | Invalid | AA |
| 17 | IR24-J | in | GG | TT | GG | CC | CC | TT | CC | TT | AA |
| 18 | IR64 (p1) | in | GG | TT | GG | CC | CC | TT | CC | TT | AA |
| 19 | IR64 (p2) | in | GG | TT | GG | CC | CC | TT | CC | TT | AA |
| 20 | IR64 (p3) | in | GG | TT | GG | CC | CC | TT | CC | Invalid | AA |
| 21 | IRTP743 | ja | GG | TT | GG | CC | CC | TT | CC | TT | CC |
| 22 | Kasalath (p1) | in | GG | TT | GG | No call | CC | TT | CC | CC | CC |
| 23 | Kasalath (p2) | in | GG | TT | GG | CC | CC | TT | CC | CC | CC |
| 24 | Koshihikari (p1) | ja | GG | TT | GG | CC | CC | AA | CC | CC | CC |
| 25 | Koshihikari (p2) | ja | GG | TT | GG | CC | CC | AA | CC | CC | CC |
| 26 | LTH | ja | GG | TT | GG | CC | CC | AA | CC | CC | CC |
| 27 | Minghui63 | in | GG | TT | GG | CC | CC | TT | TT | TT | AA |
| 28 | Nipponbare (p1) | ja | GG | TT | GG | CC | CC | AA | CC | CC | CC |
| 29 | Nipponbare (p2) | ja | GG | TT | GG | CC | CC | AA | CC | CC | CC |
| 30 | NIL1-3-12 | in | GG | TT | GG | CC | CC | AA | CC | CT | AC |
| 31 | *O. alta* | WS | No call | No call | No call | No call | CC | AA | No call | No call | CC |
| 32 | *O. longistaminata* | WS | GG | TT | GG | CC | CC | TT | CC | CC | CC |
| 33 | Osmancik | ja | GG | TT | GG | CC | CC | AA | CC | No call | CC |
| 34 | NSIC Rc158 | in | GG | TT | GG | CC | CC | TT | CC | TT | AA |
| 35 | NSIC Rc222 | in | TT | AA | AA | CC | CC | TT | TT | TT | AA |
| 36 | NSIC Rc238 | in | GG | TT | GG | CC | CC | TT | TT | TT | AA |
| 37 | PSB Rc82 | in | GG | TT | GG | CC | CC | TT | TT | TT | AA |
| 38 | Sabita | LA | GG | TT | GG | CC | CC | TT | CC | TT | AA |
| 39 | Sadutcho | ja | GG | TT | GG | CC | CC | TT | CC | Invalid | CC |
| 40 | Sasanishiki | ja | GG | TT | GG | CC | CC | TT | CC | TT | CC |
| 41 | ST12 (p1) | in | TT | AA | AA | TT | CC | TT | TT | TT | AA |
| 42 | ST12 (p1) | in | TT | AA | AA | TT | CC | TT | TT | TT | AA |
| 43 | ST6 | ja | TT | AA | AA | CC | CC | TT | CC | CC | CC |
| 44 | Tequing-12 | in | TT | AA | AA | CC | CC | TT | CC | Invalid | CC |
| 45 | Tequing-93 | in | TT | AA | AA | CC | CC | TT | CC | TT | CC |
| 46 | TN1-105 | ja | GG | TT | GG | CC | CC | TT | CC | Invalid | CC |
| 47 | TN1-45 | ja | GG | TT | GG | CC | CC | TT | CC | No call | CC |

^a^ When more than two plants from the same accession were analyzed, the plant number was given as (p).

^b^ in, *indica*; ja, *japonica*; WS, wild rice species; LA, landrace; No call, no flourescence signal
