# Supplementary material for: Development and validation of allele-specific SNP/indel markers for eight yield-enhancing genes using whole-genome sequencing strategy to increase yield potential of rice, Oryza sativa L
Source: Rice (N Y). 2016 Mar 18;9:12. doi: 10.1186/s12284-016-0084-7 (PMC4797370; doi:10.1186/s12284-016-0084-7)
Supplement: Additional file 6: Table S2. — Summary of whole-genome sequencing of six varieties. (DOC 47 kb) [file 12284_2016_84_MOESM6_ESM.doc]

**Additional file 6: Table S2.** Summary of whole-genome sequencing of six varieties

| **Nipponbare** |  |  |  |  |  |  |  |  |
| --- | --- | --- | --- | --- | --- | --- | --- | --- |
| ID | Total no. of reads | Mapped reads | | |  | Uniquely mapped reads | | |
|  |  | No. | % | Coverage (%) |  | # | % | Coverage (%) |
| Habataki | 111,938,792 | 55,084,103 | 49.21 | 14.92 |  | 45,002,095 | 40.20 | 0.00000011 |
| ST6 | 105,222,324 | 60,703,369 | 57.69 | 14.02 |  | 52,565,529 | 49.96 | 0.00000013 |
| ST12 | 104,982,210 | 57,344,799 | 54.62 | 13.99 |  | 46,726,018 | 44.51 | 0.00000012 |
| NSIC Rc158 | 90,709,988 | 44,348,057 | 48.89 | 12.09 |  | 35,811,557 | 39.48 | 0.00000010 |
| NSIC Rc222 | 98,568,814 | 48,074,091 | 48.77 | 13.13 |  | 38,771,979 | 39.33 | 0.00000010 |
| NSIC Rc238 | 97,879,364 | 46,638,754 | 47.65 | 13.04 |  | 37,753,758 | 38.57 | 0.00000010 |
| Total | 609,301,492 | 312,193,173 |  |  |  | 256,630,936 |  |  |
|  |  |  |  |  |  |  |  |  |
| **93-11** |  |  |  |  |  |  |  |  |
| ID | Total no. of reads | Mapped reads | | |  | Uniquely mapped reads | | |
|  |  | No. | % | Coverage (%) |  | # | % | Coverage (%) |
| Habataki | 111,938,792 | 62,712,102 | 56.02 | 15.22 |  | 54,796,324 | 48.95 | 0.00000013 |
| ST6 | 105,222,324 | 52,917,578 | 50.29 | 14.30 |  | 45,799,558 | 43.53 | 0.00000011 |
| ST12 | 104,982,210 | 61,335,257 | 58.42 | 14.27 |  | 53,008,911 | 50.49 | 0.00000013 |
| NSIC Rc158 | 90,709,988 | 49,947,757 | 55.06 | 12.33 |  | 43,361,854 | 47.80 | 0.00000013 |
| NSIC Rc222 | 98,568,814 | 55,132,548 | 55.93 | 13.40 |  | 47,916,015 | 48.61 | 0.00000013 |
| NSIC Rc238 | 97,879,364 | 53,556,508 | 54.72 | 13.31 |  | 46,714,482 | 47.73 | 0.00000013 |
| Total | 609,301,492 | 335,601,750 |  |  |  | 291,597,144 |  |  |
