# Supplementary material for: Development and validation of allele-specific SNP/indel markers for eight yield-enhancing genes using whole-genome sequencing strategy to increase yield potential of rice, Oryza sativa L
Source: Rice (N Y). 2016 Mar 18;9:12. doi: 10.1186/s12284-016-0084-7 (PMC4797370; doi:10.1186/s12284-016-0084-7)
Supplement: Additional file 7: Table S3. — Primers for preparation of PCR products and PCR product sequencing. (DOC 35 kb) [file 12284_2016_84_MOESM7_ESM.doc]

**Additional file 7: Table S3.** Primers for preparation of PCR products and PCR product sequencing

| Gene | Forward/ reverse | Forward primer sequence | Reverse primer sequence | Location^a^ | Product size (bp) |
| --- | --- | --- | --- | --- | --- |
| *Gn1a* | Gn1a-F1 / R1 | GGTAATGGCACACTATGCAG | CTTGACGAAGCAGTTGAGCA | promoter - 5' UTR region | 1198 |
| *OsSPL14* | SPL14-F1 / R1 | TAGCCATAGCTTCTGCGTGA | GCACACCGTTGACAAGTGAT | promoter region (4.4- 3.8 kb) | 610 |
| *OsSPL14* | SPL14-F3 / R3 | CTTTGGCATCACGCTACGGT | GATCGATGGCAGCTGGAACA | third exon | 839 |
| *SCM2* | SCM2-F1 / R1 | GCAGAGAGAAGATGCGTTGG | CTCAGCTCTGATCTGTTATTC | promoter region (1.1- 0.4 kb) | 693 |
| *SCM2* | SCM2-F3 / R3 | CCATTGATGACGCATGGGTA | ACCATCATGCATGCCATGCA | third exon | 651 |
| *SCM2* | SCM2-F4 / R4 | TGGTGTGAAGGCCAAACTCT | TCTTCGAATGATGCGTTCCAA | promoter region (3.2- 1.9 kb) | 1364 |
| *Ghd7* | Ghd7-F1 / R1 | GACCTCACCTGCTATAGCTA | GCTCGATCGAGCCGATCAT | first exon | 580 |
| *Ghd7* | Ghd7-F2 / R2 | TGCTTATGCGTACATCTGGAT | GTTAGTGGTATATACGCACTGT | second exon | 463 |
| *GS5* | GS5-F1 / R1 | CCTATCTAATCTTGACTGATTCC | GGAAAGCGAAACTGATTGACA | promoter region (1.6- 0.3 kb) | 1368 |

^a^ For the promoter region, the locations of the forward primer and the reverse primer represented the distance from the translation initiation codon.
