# Supplementary material for: Development and validation of allele-specific SNP/indel markers for eight yield-enhancing genes using whole-genome sequencing strategy to increase yield potential of rice, Oryza sativa L
Source: Rice (N Y). 2016 Mar 18;9:12. doi: 10.1186/s12284-016-0084-7 (PMC4797370; doi:10.1186/s12284-016-0084-7)
Supplement: Additional file 8: Table S4. — Markers for Fluidigm SNP genotyping platform. (DOC 36 kb) [file 12284_2016_84_MOESM8_ESM.doc]

**Additional file 8: Table S4.** **Markers for Fluidigm SNP genotyping platform**

| Marker | Allele1 (FAM) | Allele 2 (HEX) | Allele1-specific primer | Allele2-specific primer | Locus-specific primer | Specific target amplification primer |
| --- | --- | --- | --- | --- | --- | --- |
| Gn1a-19SNP-FD | G | T | CTCATCCTCTTATGGGATTCTCTATGG | ACTCATCCTCTTATGGGATTCTCTATGT | GCCCTTGCATCCCTACCATG | AGACGTGCAATCTCTCTATACCAC |
| Gn1a-18SNP-FD | T | A | CATCCGTCTCAAAAAGGATGTGT | CATCCGTCTCAAAAAGGATGTGA | AGCTTTCTCAAATTAATTAGTTATTAGGTTCTAGGTATAA | GGGCTAACGACGACTATTGC |
| Gn1a-17SNP-FD | G | A | CATACCTAGCGTTCTATGCGGG | CATACCTAGCGTTCTATGCGGA | AACCAGTAAACTTAAAATGGAAGATAAAGAAATTTCACA | GGTGAACTAATTGGCTGAACATACC |
| SPL14-04SNP-FD | C | T | CGTGCTTACCGCCGGG | CGTGCTTACCGCCGGA | CTCTAGTAGCCTATCCACATACCAGGA | ACCACGTGGGAACCGT |
| SPL14-12SNP-FD | C | A | CCGACTCGAGCTGTGCTC | CCGACTCGAGCTGTGCTA | TCCCATGGCTGGGTTGACA | CAGGTGGATGTCTCGCAG |
| Ghd7-05SNP-FD | A | T | GGGTTCAAGCTCTCCCCAT | GGGTTCAAGCTCTCCCCAA | AGCTCGATGCCCAAGGAGAT | CGCCAACCACCGTGTTT |
| GS5-01SNP-FD | T | C | CGTTTCATTATTCACGTTTCGCGATTA | GTTTCATTATTCACGTTTCGCGATTG | GGAGTAGATGCACGGTGCC | AGTCTGCCTAACTTCTTCGCT |
| GS5-03SNP-FD | T | C | ATGCGTGCCAATATTCCTGTAGTA | GCGTGCCAATATTCCTGTAGTG | CGGAACGCAGCCTAACTACC | AAAGTCTGTTTTTCACAGGTACCAT |
| GS3-01SNP-FD | C | A | CTGCCTCCAGATGCTGC | GCTGCCTCCAGATGCTGA | ACAGCAGGCTGGCTTACTCT | AAATTCAATCGAAGGGATCCACG |
